# MicroRNA-loaded biomaterials for osteogenesis

**DOI:** 10.3389/fbioe.2022.952670

**Published:** 2022-09-19

**Authors:** Jingwei Wang, Yutao Cui, He Liu, Shaorong Li, Shouye Sun, Hang Xu, Chuangang Peng, Yanbing Wang, Dankai Wu

**Affiliations:** Orthopaedic Medical Center, Second Hospital of Jilin University, Changchun, China

**Keywords:** bone defect, miRNA, biomaterial, molecular mechanisms, bone regeneration

## Abstract

The large incidence of bone defects in clinical practice increases not only the demand for advanced bone transplantation techniques but also the development of bone substitute materials. A variety of emerging bone tissue engineering materials with osteogenic induction ability are promising strategies for the design of bone substitutes. MicroRNAs (miRNAs) are a class of non-coding RNAs that regulate intracellular protein expression by targeting the non-coding region of mRNA3′-UTR to play an important role in osteogenic differentiation. Several miRNA preparations have been used to promote the osteogenic differentiation of stem cells. Therefore, multiple functional bone tissue engineering materials using miRNA as an osteogenic factor have been developed and confirmed to have critical efficacy in promoting bone repair. In this review, osteogenic intracellular signaling pathways mediated by miRNAs are introduced in detail to provide a clear understanding for future clinical treatment. We summarized the biomaterials loaded with exogenous cells engineered by miRNAs and biomaterials directly carrying miRNAs acting on endogenous stem cells and discussed their advantages and disadvantages, providing a feasible method for promoting bone regeneration. Finally, we summarized the current research deficiencies and future research directions of the miRNA-functionalized scaffold. This review provides a summary of a variety of advanced miRNA delivery system design strategies that enhance bone regeneration.

## 1 Introduction

Bone defects caused by congenital diseases, tumors, and trauma are common challenges in clinical treatment. In these cases, the repair capacity of the bone tissue is impaired, and therefore, self-healing is difficult without surgical intervention. In clinical practice, the gold standard for the treatment of bone defects is still autologous bone transplantation (Collon et al., 2021). Unfortunately, the source of autologous bone transplantation is limited, and complications, such as pain, occur at the donor site. As an alternative therapy, allogeneic bone transplantation is expensive and prone to disease transmission and rejection (Brink, 2021). Therefore, it is of great clinical significance to find new biomaterials that promote bone regeneration and solve serious problems of bone defects.

Bone tissue engineering is an interdisciplinary field that combines biological factors and material engineering to regenerate damaged bone tissue. It can be used as an alternative therapy for autografts and allografts (Gundu et al., 2022). A variety of bone tissue engineering materials loaded with growth factors have been shown to locally promote osteogenic differentiation and repair bone defects (Li et al., 2021b). However, the stability of these growth factors is poor, and bone-promoting drugs, such as bisphosphonates and simvastatin, as their substitutes, have insufficient bone-inducing efficacy (Cui et al., 2019b; Bai et al., 2020; Jin et al., 2021). In recent years, nucleic acid transfer with osteogenesis regulation has shown great potential in the treatment of bone injury, owing to its persistent local effect and low cost (Ou et al., 2019). Exogenous nucleic acid substances can enter mesenchymal stem cells through cell phagocytosis or interact with the cell surface through exogenous interference to regulate the activity of intracellular-related transcription factors and interfere with the process of cell differentiation (Kim et al., 2021). In the field of nucleic acid molecular-based bone engineering, there is consensus that the sustainable provision of appropriate carriers of nucleic acid molecules at the physiological level is essential for osteogenic efficacy.

MicroRNAs (miRNAs) are a series of small, single-stranded, non-coding RNAs (Costa et al., 2019; Chen et al., 2020b). miRNAs can bind to the 3′-UTR noncoding region of the target mRNA while completely complementing each other to inactivate and degrade the mRNA or play a negative regulatory role at the post-transcriptional level by interfering with mRNA translation, which is a key regulatory factor in cell osteogenic differentiation (Arfat et al., 2018; Tang et al., 2020). In recent years, the effect of miRNAs on the treatment of bone-related diseases has become increasingly recognized (Liu et al., 2019; Hu et al., 2020). The introduction of miRNA into osteoblast-related cells can transmit osteoblast-related information through specific signaling pathways, including bone morphogenetic proteins (BMPs)/*Drosophila* mothers against decapentaplegic proteins (SMADs)/runt-related transcription factor 2 (Runx2), Wnt/β-catenin and MAPK, regulate gene expression activity, and promote osteoblast differentiation (Li et al., 2018; Feng et al., 2020). In addition, miRNAs can regulate osteogenic differentiation by interacting with other non-coding RNAs, such as lncRNAs and circRNAs. Some specific lncRNAs and circRNAs can be used as competitive endogenous RNAs (ceRNAs) of miRNAs or sponges of specific miRNAs to reduce the function of miRNAs and act as miRNA inhibitors (Yuan et al., 2019b; Peng et al., 2019).

However, it is difficult for miRNAs to directly act on the osteogenic microenvironment due to the fact that negatively charged miRNAs are difficult to penetrate positively charged cell membranes under the effect of Coulombic force, and their single-stranded structure is unstable, which is easy to be degraded by RNase (Cui et al., 2019a; Ou et al., 2019). The design and synthesis of small double-stranded miRNAs using mature miRNAs as templates—miRNA mimics and miRNA agomir—can better solve these problems (Wang et al., 2018b). These double-stranded miRNAs have a more stable molecular structure and can play a larger role when transfected into cells (Yu et al., 2020). Therefore, the combination of miRNA mimics and miRNA agomir with a variety of biomaterials as factors regulating the local osteogenic microenvironment has been the subject of a large number of studies in recent years. A large number of experiments have confirmed that miRNA-loaded biomaterials exhibit excellent biosafety and effectively promote the healing of bone defects (Ma et al., 2020a; Sun et al., 2020b; Bu et al., 2020).

**SCHEME 1:**
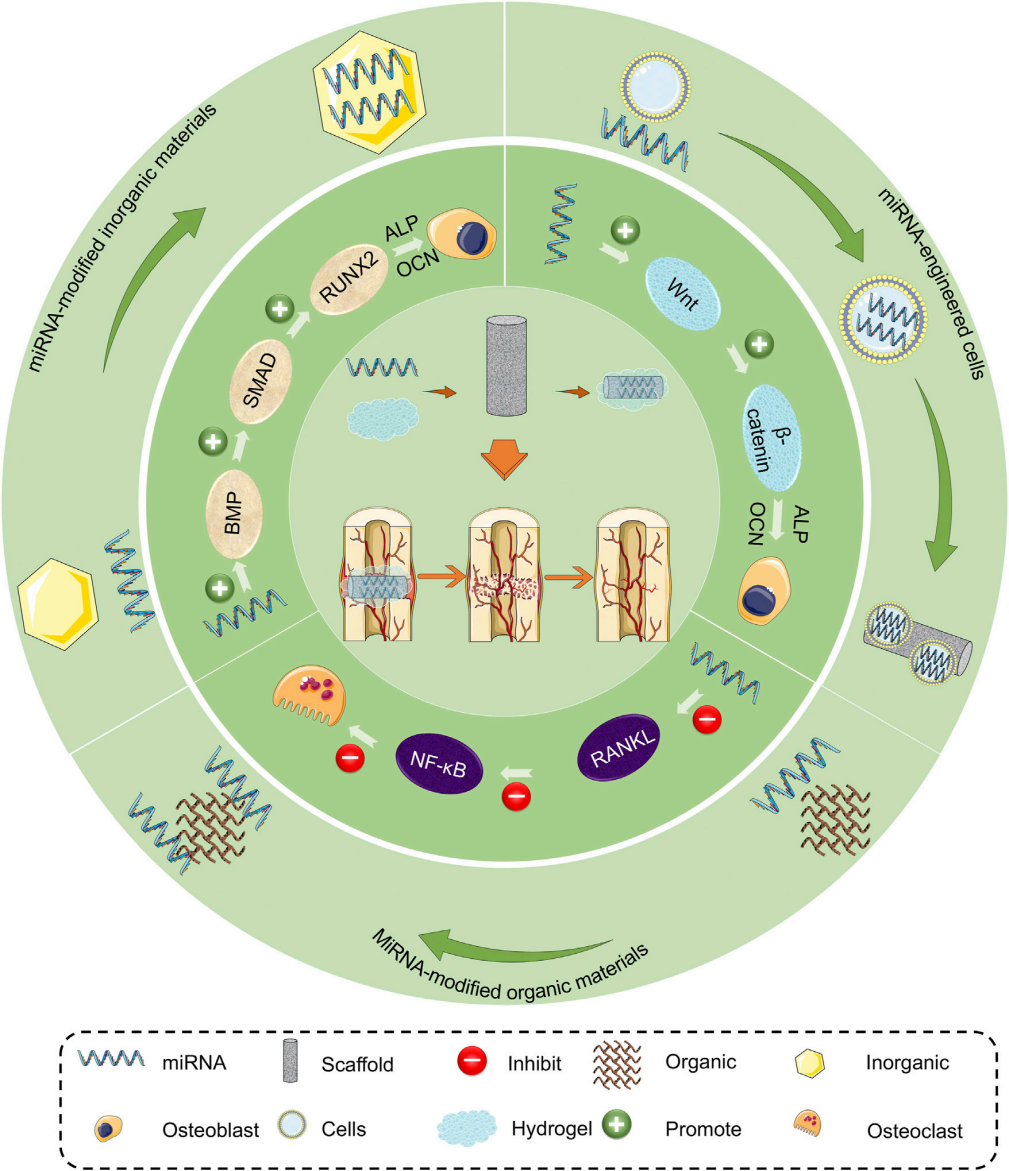
Mechanism and application of miRNAs promoting bone regeneration.

In this review, we analyze the molecular mechanisms of miRNA regulating osteogenic differentiation of mesenchymal stem cells to provide a comprehensive understanding of their application. The application of miRNA-loaded biomaterials in promoting bone regeneration in bone defects is summarized, and possible treatment is discussed (Scheme 1). Finally, we present the advantages and disadvantages of various miRNA-loaded biomaterials, which provide a theoretical basis and advanced methods for the treatment of bone defects.

## 2 Mechanism of miRNAs in bone regeneration

miRNAs can interfere with the differentiation of MSCs by participating in multiple signaling pathways and targeting bioactive factors involved, including BMPs/SMADs/RUNX2, Wnt/β-catenin, MAPK, and phosphatase, and the tensin homolog deleted on chromosome 10 (PTEN)/phosphoinositide 3-kinase (PI3K)/AKT (Li et al., 2018; Jia et al., 2019; Sun et al., 2020a; Shi et al., 2020). In this section, we summarize and discuss a variety of osteogenesis-related pathways regulated by miRNA, which provide a theoretical basis for the use of miRNAs to promote bone regeneration.

### 2.1 BMPs/SMADs/RUNX2

The BMP signaling pathway plays an important role in bone growth and development, and its specific expression is key to the osteogenic differentiation of stem cells (Kaur et al., 2018; De Mattei et al., 2020). The key proteins of the BMP signaling pathway are BMP proteins and BMP receptors (BMPRs), SMAD proteins, and the Runx2 factor (Yin et al., 2018; Shen et al., 2020). These three proteins are also the target proteins of most miRNA molecules. BMPs are a series of special proteins synthesized by osteoblasts and secreted into the bone matrix microenvironment (Xiong et al., 2020b; Cao et al., 2020). They can bind to BMPR and phosphorylate them and then activate the downstream SMAD signaling molecules to promote osteogenic differentiation (Zhang et al., 2017a; Peng et al., 2019; Wang et al., 2020c). miRNAs can directly regulate the mRNA expression of BMP and its receptor to regulate the osteogenic differentiation of cells. MiR-765 and miR-451a inhibit osteogenic differentiation of mesenchymal stem cells by specific recognition and binding to BMP-6 mRNA (Lu et al., 2019; Wang et al., 2020c). MiR-98 binds to BMP2 mRNA and inhibits osteogenic differentiation of BMSCs (Zhang et al., 2017a; Zhi et al., 2021). In addition, miRNA can also indirectly regulate the expression of BMPs by affecting the activity of related proteins that regulate BMPs. For example, noggin is a specific inhibitor of BMP2, and miR-148b effectively promotes osteogenic differentiation of BMSCs by targeting noggin (Li et al., 2017; Akkouch et al., 2019). In addition to regulating the noggin that reduces the BMP activity, miRNA can also target promoter-binding proteins of DNA that encodes BMPs. For example, LSD1 demethylates the promoter histone lysine, and miR-137 indirectly promotes osteogenic differentiation by targeting LSD1 mRNA (Ma et al., 2020b). There are also some miRNAs that negatively regulate bone formation by interacting with BMP receptors. Studies confirmed that a variety of miRNAs can act on BMPR2, thereby affecting the activation of downstream proteins, such as SMAD, and thus inhibiting osteogenesis (Vishal et al., 2017; Luo et al., 2018; Hao et al., 2021).

SMAD1/5/8 is activated by the BMP receptor and transmits a BMP signal. SMAD4 can form complexes with activated SMAD1/5/8 to regulate osteogenic gene expression in the nucleus with heterodimeric complexes of multiple transcription factors (co-activators or co-inhibitors). Meanwhile, this process can be blocked by inhibitory SMAD, such as SMAD-6 and SMAD-7 (Vishal et al., 2017; Luo et al., 2018; Hao et al., 2021). SMAD4 and SMAD7 are also important target proteins of miRNA. MiR-144-3p and miR-224 inhibit SMAD4 expression by targeting SMAD4 mRNA 3′UTR (Luo et al., 2018). Valenti et al. (Vishal et al., 2017; Yang et al., 2019; Smieszek et al., 2020) found that miR-21 targets SMAD7 and increases RUNX2 transcription, which has an anti-apoptotic effect on bone marrow mesenchymal stem cells and promotes their osteogenic potential.

The activated SMADs interact physically with Runx2 and protect RUNX2 protein from degradation (Vishal et al., 2017; Srinaath et al., 2019). Runx2 is the main regulator of osteogenesis, which will promote the expression of osteopontin (OPN) and osteocalcin (OCN) (Kureel et al., 2017; Hu et al., 2020; Marycz et al., 2021). Some miRNAs directly target RUNX2 to regulate the BMP/SMAD signaling pathway and interfere with other signaling pathways (Zhang et al., 2017c; Huang et al., 2020a). MiR-133a/135a/93-3p targets Runx2 mRNA to inhibit the differentiation process of osteoblast cells (Zhang et al., 2017a; Kureel et al., 2017). RUNX2 can also be indirectly promoted by miRNAs, by targeting mRNA of RUNX2-inhibiting molecules. For instance, HDAC4 is a conserved enzyme that removes the lysine side chain acetyl on histone and regulates bone formation by inhibiting the RUNX2 expression activity (Wang et al., 2020b). MiR-30c/29 protects the RUNX2 molecule by targeting HDAC4 and inhibiting its activation (Lian et al., 2019; Srinaath et al., 2019). The E1A-binding protein P300 (EP300) inhibits the activity and acetylation of RUNX2, which in turn inhibits osteoblast differentiation and mineralization. Overexpression of miR-132-3p and miR-22-3p reverses this process by inhibiting protein translation of EP300 (Hu et al., 2015; Makitie et al., 2018). Inhibiting the translation of RUNX2 activator protein is also a method for miRNA to regulate RUNX2 activity (Kureel et al., 2017; Godfrey et al., 2018). MiR-124/141 inhibits RUNX2 activation by targeting Dlx4 mRNA, an activator of Runx2, thereby inhibiting OCN and OPN (Kureel et al., 2017; Marycz et al., 2021). The miRNAs targeting other key molecules in the BMP/SMAD/RUNX2 signaling pathway are summarized in Table 1.

**TABLE 1:** Summary of target genes of different signaling pathways by miRNAs.

Signaling pathways	Target gene	miRNA	Cell type	Effect on osteogenesis
BMP/SMAD	SMAD2	miR-10b	HADSCs	Promotion
	BMP-7	miR-542-3p	Calvarial osteoblasts	Inhibition
	BMP2	miR-6979-5p/214	MC3T3-E1	Inhibition
	BMPR	miR-125b	HBMSCs	Inhibition
	RUNX2	miR-203	HBMSCs	Inhibition
	SMAD7	miR-877-3p/15b/590-5p	HBMSCs	Promotion
	SMAD4	miR-664-3p	MC3T3-E1	Inhibition
	SMAD5	miR-135/25-3p	hUC-MSCs	Inhibition
	Dlx5	miR-141/200a	MC3T3-E1	Inhibition
	Noggin	miR-200c	HBMSCs	Promotion
Wnt/β-catenin	Wnt2b	miR-370-5p	HBMSCs	Inhibition
	Wnt7a	miR-889	BMMSCs	Inhibition
	Wnt3a	miR-503	MC3T3-E1	Inhibition
	Wnt1	miR-22-3p/miR-34a-5p	—	Inhibition
	Wnt10b	miR-33b-5p	hUC-MSCs	Inhibition
	Wnt11	miR-154-5p	ADSC	Inhibition
	DKK-3	miR-129-5p	BMSCs	Promotion
	APC	miR-27a/675	HBMSCs	Inhibition
	GSK-3β	miR-26a	BMSCs	Promotion
	DKK1/Kremen2	miR-291a-3p	BMSCs	Promotion
	PPARγ	miR-381/130a/27a	BMSCs	Promotion
	LRP6	miR-30e	Preadipocyte 3T3-L1	Inhibition
	LRP5	miR-23a	BMSCs	Inhibition
PTEN/PI3K/AKT	PTEN	miR-19b/214/21	MC3T3-E1	Promotion
MAPK	FGF2	miR-16	BMSC	Inhibition

### 2.2 Wnt/β-catenin

The Wnt/β-catenin signaling pathway plays a complex role in osteogenic differentiation (Luo et al., 2019; Luo et al., 2020). Wnt protein is the starting molecule of the Wnt/β-catenin signaling pathway, which can be inhibited by miRNA. Wnt6 and Wnt10a mRNAs were identified as targets of miR-378 (Feng et al., 2020); miR-378 inactivates the signal by inhibiting their expression and ultimately delays bone formation. Reducing the expression of these miRNAs is a feasible way to strengthen Wnt/β-catenin signaling. Studies have shown that miR-503-3p decreased during distraction of osteogenesis, increased the transcription of targeted Wnt2 and Wnt7b, and activated the Wnt/β-catenin signaling pathway (Luo et al., 2020). The LDL receptor-related protein 5 (LRP5) is one of the Wnt protein receptors. miRNAs targeting LRP5 will make the activated Wnt protein unable to stimulate the dishevelled (Dsh or Dvl) in the downstream cytoplasm and thus inhibit signal transduction (Ma et al., 2020a).

The Wnt pathway is also regulated by miRNAs acting on pathway inhibitors, such as GSK-3β and DKK-1 (Wu et al., 2021). Upstream activation of Dvl leads to the dissolution of the downstream β-catenin degradation complexes, including APC and GSK-3β, to avoid the ubiquitination of β-catenin by complexes and transport to the proteasome for degradation (Ma et al., 2020a; Yang et al., 2020). Under different induction conditions, miR-138 regulates osteoblast proliferation, differentiation, adhesion, and apoptosis by targeting Dvl2 and GSK-3β, respectively (Chen et al., 2021). Furthermore, DKK family proteins, another kind of classic Wnt signaling pathway inhibitors, are also the target of miRNA regulation of osteogenesis. DKK family proteins are cysteine-rich secreted proteins that bind the Kremen protein and Wnt co-receptor LRP5 to form trimers to mediate intracellular phagocytosis, reduce Wnt receptors on the cell surface, and antagonize Wnt signal transduction (Zhang et al., 2017b). MiR-29a was found to target DKK-1 to activate osteogenic gene expression and matrix mineralization, and promote bone formation (Wu et al., 2021). MiR-335-5p and miR-217 can also directly bind to DKK1 mRNA3′-UTR to activate Wnt signaling (Zhang et al., 2017b; Dai et al., 2019).

The regulation of the β-catenin activity is the final key to the Wnt/β-catenin pathway signal transduction. Lowering the expression level of β-catenin is key to this regulation. CTNNB1 is a β-catenin encoding gene that directly regulates the β-catenin expression level. MiR-320a was shown to directly target CTNNB1 mRNA (Wang et al., 2020a). TCF7-encoded protein forms a complex with β-catenin and activates transcription through the Wnt/β-catenin signaling pathway. MiR-22-3p and miR-138-5p indirectly affect the β-catenin activity by targeting TCF7 mRNA (Makitie et al., 2018; Chen et al., 2021). Moreover, it is equally important to regulate the inhibitor activity of β-catenin. E-cadherin can stimulate β-catenin nuclear translocation and transcription by stimulating LRP from the cell surface to cytoplasm, which plays a regulatory role in maintaining the β-catenin structural integrity and function (Feng et al., 2018). ZEB2 is a negative regulator of E-cadherin and is targeted by miR-138/145 (Feng et al., 2018). It was reported that peroxisome proliferator-activated receptor γ (PPAR-γ) inhibits Wnt signaling by targeting phosphorylation of β-catenin (Liu et al., 2006). MiR-381/130a/27a targeting PPAR-γ protects β-catenin from osteogenic differentiation and inhibits lipid differentiation (Lin et al., 2019; Long et al., 2019; Hao et al., 2021). The target gene of Let-7i-3p is lymphatic enhancement factor 1 (LEF1) mRNA (Luo et al., 2019). In the activated Wnt signaling pathway, LEF1 forms dimers with over-aggregated β-catenin. Then, these complexes enter the nucleus and promote gene transcription, thus playing a regulatory role in signal transduction. The microRNAs targeting the Wnt/β-catenin signaling pathway are listed in Table 1.

### 2.3 RANKL/NF-κB

Normal bone metabolism involves a dynamic balance between osteoblast-mediated bone formation and osteoclast-mediated bone resorption. However, in some pathological states, such as osteoporosis and osteomyelitis, the local osteoclastic activity of bone defects can be very strong, resulting in bone damage. Therefore, in these cases, regulating the activity of osteoclasts is also crucial for promoting bone defect repair. The macrophage colony-stimulating factor (M-CSF) produced by osteoblasts induces the expression of the NF-κB receptor activator (RANK) on the surface of bone marrow-derived monocyte precursors and then combines with the RANK ligand (RANKL) to promote the expression of NF-κB in cells, such that monocytes differentiate into multinuclear trap-positive osteoclasts (Guo et al., 2019). Protein arginine methyltransferase 3 (PRMT3) is activated by RANKL and acts on the promoter histone H4R3me2a encoding miR-3648, which upregulates the expression of miR-3648. MiR-3648 targeting APC2 promotes osteoclast proliferation (Zhang et al., 2019). MiR-186 positively regulates the nuclear localization and acetylation of NF-κB by targeting SIRT6, which is one of the deacetylases of H3K9 on the promoter (Xiao et al., 2020). In addition, indirect regulation of NF-κB expression is also important in influencing osteoclast differentiation. MiR-29a indirectly inhibits NF-κB activation by targeting RANKL mRNA3′-UTR and causing RANKL deficiency (Lian et al., 2019). TNFR1 is another activator of NF-κB. MiR-218 indirectly inhibits NF-κB by targeting TNFR1 and reduces bone loss induced by osteoclast differentiation (Wang et al., 2018a).

Osteoprotegerin (OPG), also known as the osteoclastogenesis inhibitor, is likewise produced by osteoblasts, which combine with RANK to interfere with RANKL localization in cells and inhibit osteoclastogenesis (Marycz et al., 2021; Wu et al., 2021). MiR-27a interferes with the localization of RANKL in cells by increasing OPG transcription, regulating the RANKL/OPG ratio, reducing osteoclast formation, and promoting bone remodeling (Guo et al., 2019).

### 2.4 PTEN/PI3K/AKT

The PTEN/PI3K/AKT signaling pathway is widely considered a tumor suppressor pathway; however, in recent years, it has been found that this signaling pathway is also closely related to osteoblast formation (You et al., 2021). There is evidence that PTEN and BMP2 function mutually antagonistically; activated PI3K/AKT is very important for BMP2-induced osteoblast differentiation (Dong et al., 2021). In addition, PI3K plays an important regulatory role in osteoclast differentiation and functional expression (Gyori et al., 2014). PTEN and AKT are key molecules of this signaling pathway. MiR-130b/26a dephosphorylates PIP3 by targeting PTEN and encodes PIP phosphatase during protosilicate-induced osteogenic differentiation of MSCs, thereby promoting AKT phosphorylation and its downstream signaling (Xiong et al., 2019). MiR-21 promotes the migration and osteogenic differentiation of BMSCs by promoting downstream expression of hypoxia-inducible factor-1 α (HIF-1α) by targeting AKT (Zhang et al., 2019; Xiao et al., 2020). Furthermore, the mammalian target of rapamycin (mTOR) is likewise downstream of AKT, and DEPTOR is an inhibitor of mTOR (Chen et al., 2017). MiR-375 inhibits mTOR expression by targeting DEPTOR, leading to activation of S6K and negative feedback activation of IRS1-PI3K-AKT, which leads to a reduction of phosphorylated AKT molecules and promotes osteogenic differentiation (Chen et al., 2017).

### 2.5 MAPK pathway

ERK, a member of MAPK, is considered an indispensable key molecule in osteogenic differentiation. FGF2 is an upstream activator of MAPK/ERK and plays a primary role in ERK activity regulation (Soszyńska et al., 2019). According to Mi et al. (2020), miR-7212-5p targets the FGF2 receptor, thereby indirectly inhibiting downstream FGF2 signaling activation and osteogenic differentiation.

Activated ERK affects the expression activity of the Hippo signaling pathway. After activation of the Hippo signaling pathway, it is subjected to a series of phosphorylation reactions of kinases and eventually acts on downstream effector factors YAP and TAZ. Therefore, the Hippo signal activity was mainly affected by the ratio of YAP1 to TAZ and plays a role in organ development, tissue regeneration, and functional differentiation of stem cells (Chen et al., 2017). MiR-33a-3p and miR-375 interfere with cell proliferation and apoptosis by targeting YAP1 to change the ratio of YAP1 to TAZ (Chen et al., 2017; Costa et al., 2019). MiR-214 and miR-376b-3p can also target YAP1; however, this process can be absorbed by circ-ITCH and circ-0024097 sponges, respectively (Huang et al., 2020b; Zhong et al., 2021).

## 3 Application of miRNA-loaded biomaterials in bone regeneration

MiRNA can regulate cell osteogenic differentiation through multiple signaling pathways, giving it good application prospects in the bone defect area. In recent studies, the application of miRNA in promoting bone defects can be divided into two strategies, namely, the construction of engineered cells by transfecting miRNA into cells *in vitro* (to promote osteogenesis by transplanting exogenous engineered osteoblasts to the bone defect area) and the direct modification of biological materials by miRNA (to regulate osteogenic differentiation of host cells through the local release of miRNA). This section summarizes and discusses these two perspectives and is summarized in Table 2.

**TABLE 2:** Summary of advantages and disadvantages of various biological materials.

Materials	Advantages	Disadvantages
Viral vectors	High transfection characteristics	Potential genotoxicity and immune inflammatory response
Exosomes	Protect miRNAs from extracellular environmental pollution or degradation and does not cause immune rejection	Different subtypes of exosomes may lead to different outcomes
Plasmid	High transfection efficiency and cell innocuity	Unstable structure and low lysosome escape rate
CS	Biocompatibility and antibacterial property	Transshipment capacity is limited, and metabolism is difficult
PEG	Excellent softness and water solubility	Excessive degradation and lacks of pharmacokinetic stability
PLGA	Strong biological binding ability and structural controllability	Degradation of products easily affects cell activity
PEI	Enhancement of cell adhesion, aggregation, and osteogenic activity	Effect of osteogenesis is not significant at low concentrations and has strong biotoxicity at high concentrations
Metallic materials	Good mechanical and antibacterial properties	Lack of sufficient carrying capacity
Calcium orthophosphate	Good biocompatibility and osteogenic induction	Mechanical brittleness
Silicon-based materials	Degradation nontoxic	Lack of sufficient nucleic acid-binding ability

### 3.1 miRNA-engineered cells with the enhanced osteogenic ability

#### 3.1.1 Engineered cells mediated by viral vectors

Because of their unique high transfection characteristics, viruses easily penetrate the cell membrane to reach the cells and integrate their own genetic material into the nucleus to express the target gene (Ou et al., 2019). Therefore, by introducing a gene encoding a miRNA capable of promoting osteogenesis into osteogenic-related cells through a viral vector, engineered cells with a persistently high expression of osteogenic markers can be obtained.

**FIGURE 1:**
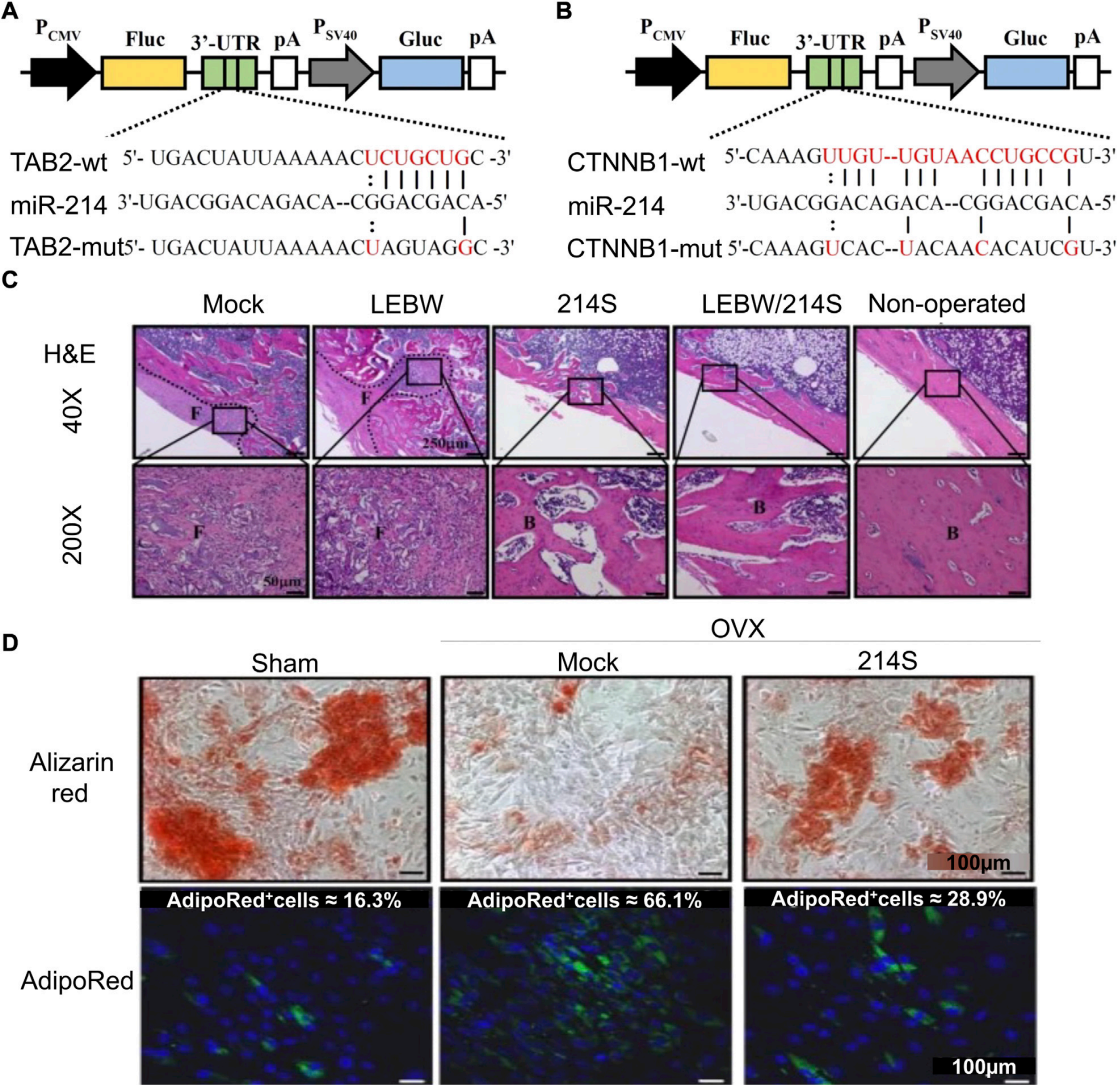
Mixed baculovirus carrying the miR-214 sponge sequence promotes osteogenic differentiation of ASCs (Li et al., 2017). **(A,B)** Schematic diagrams of four reporter gene plasmids were constructed to express Gaussia luciferase (Gluc) and firefly luciferase (Fluc). At the 3′-UTR site of Fluc, there were wild-type or mutant TAB2 (TAB2-wt or TAB2-mut) or CTNNB1 (CTNNB1-wt or CTNNB1-mut) sequences. **(C)** H&E staining was performed 5 weeks after femoral epiphyseal segment implantation in rats. **(D)** Alizarin red staining (ARS) and AdipoRed staining after virus transfection of adipose stem cells from ovariectomized rats (OVX-ASCs). (Mock, transduced without BV; LEBW, sustained BMP2 expression; 214S, co-transduction of OVX-ASCs with BacECre/Bac214S; Non-operated, rats without ovariectomy; Sham, ASCs were isolated from animals without OVX; H&E, hematoxylin and eosin.) Copyright 2017. Reproduced with permission from Springer Nature.

Li et al. (2017) developed a mixed baculovirus vector based on Cre/loxp. The nucleic acid sequence encoding the miR-214 sponge was added to the virus sequence to maintain its continuously high expression in cells, downregulate the level of miR-214 in cells, and then activate the target molecules β-catenin and TAB2 of miR-214 in the Wnt pathway. The upregulation of osteogenic factor β-catenin/Runx2 and downregulation of adipogenic factors PPAR-γ and C/EBP-α enhance the osteogenic ability of BMSCs (Figure 1). Yang et al. (2020) constructed ADSC-engineered cells with miR-130a-3p overexpression through lentiviral vectors. The genetically modified ADSC showed good osteogenic differentiation ability. These engineered cells can be combined with biomaterials and applied to bone defects to facilitate bone repair. Xiong et al. (2020a) reported bone bioengineering scaffolds using polyhedral polysiloxane (POSS) to enhance poly-l-lactic acid (PLLA) mechanical properties and cell adhesion. This scaffold was used for lentivirus-transfected BMSCs with high miR-19b-3p expression. Combining lentivirus-modified cells with PLLA/POSS scaffolds as a strategy of bone tissue engineering has a good effect on bone regeneration.

Unfortunately, gene insertion may cause silencing after gene mutation and even affect the expression of other genes, which is an unresolved problem, thus being considered unreliable vectors (Ou et al., 2019; Bu et al., 2020; Gan et al., 2021; Hosseinpour et al., 2021). Therefore, the harmless treatment of viral nucleic acid sequences is key to promoting its future clinical applications.

#### 3.1.2 Engineered cells mediated by transfected exosomes

**FIGURE 2:**
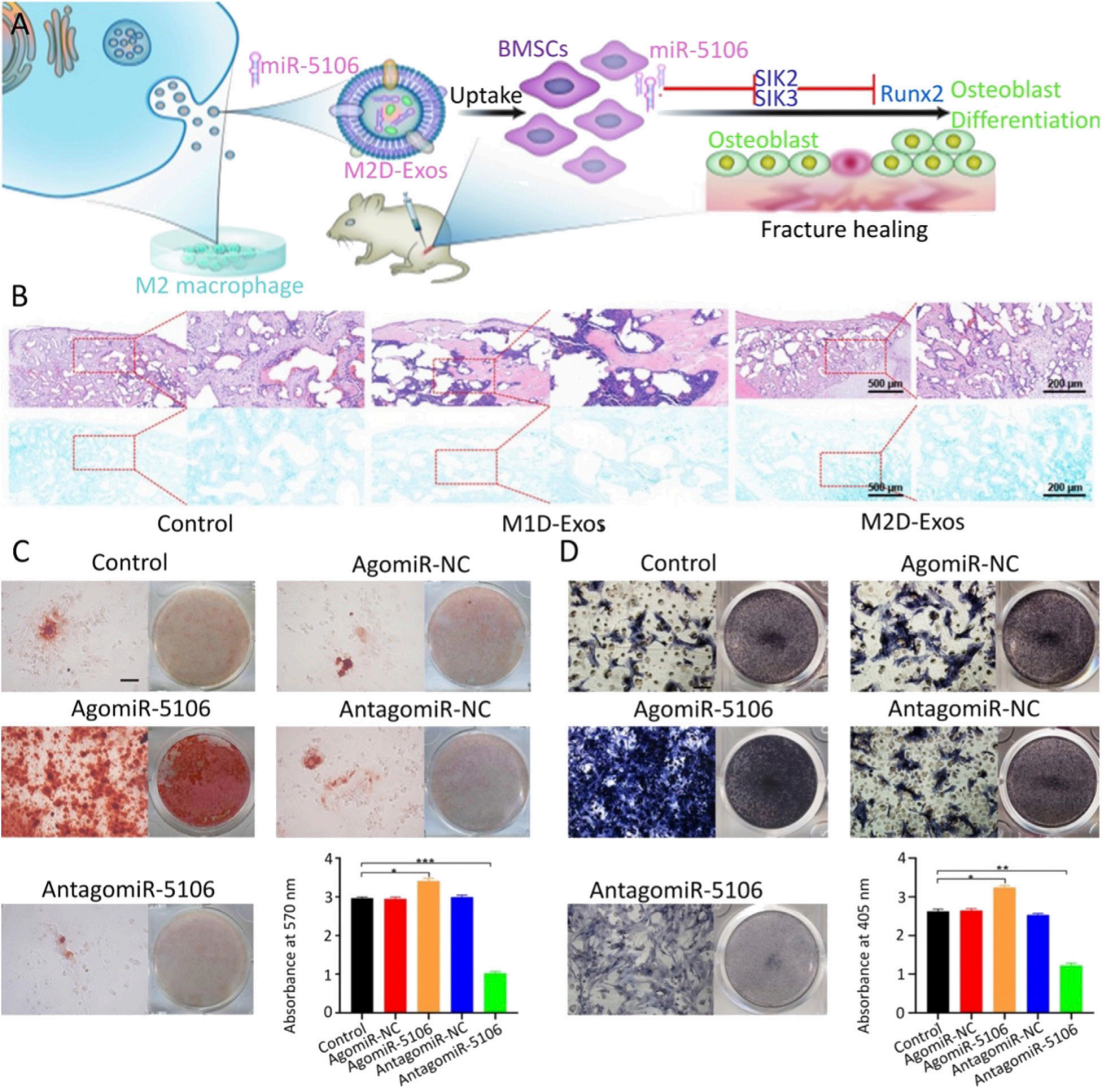
M2 macrophage-derived exosomes promote fracture healing by overexpression of miR-5106 (Xiong et al., 2020c). **(A)** Schematic diagram of M2 macrophage-derived exosomes (M2D-Exos) promoting fracture healing. **(B)** Bone tissue samples were stained with H&E/Alcian-blue staining after 21 days of treatment with PBS (control), M1 macrophage-derived exosomes (M1D-Exos), and M2D-Exos, respectively. ARS and its quantification **(C)** and alkaline phosphatase staining and its quantification **(D)** of BMSCs transfected with PBS, control agomir construct (agomir-NC), agomiR-5106, control antagomir construct (antagomir-NC), or antagomir-5106, respectively, for 21 days. Copyright 2020. Reproduced with permission from Springer Nature.

Exosomes are liposomes encapsulated by phospholipid bilayers that are secreted by cells and capable of transmitting intercellular information (Hu et al., 2021; Kim et al., 2021; Xun et al., 2021). Exosomes can bind to the cell membrane using their fluidity after contacting the surface of stem cells and then release the nucleic acids encapsulated therein into the cells (Chen et al., 2019; Zuo et al., 2019). As a carrier of nucleic acid transmission, it can protect miRNA from extracellular environmental pollution or degradation and does not cause immune rejection, making it an ideal carrier for miRNA transfection (Xu et al., 2020; Xia et al., 2021; Xun et al., 2021). Xiong et al. (2020c) found that miR-5106 was highly expressed in exosomes derived from M2 macrophages. The transfection of BMSCs with this exosome showed enhanced osteogenic activity and promoted fracture healing. In addition to its highly effective role in promoting stem cell osteogenesis, surprisingly, the transfection with M2 macrophage-derived exosomes also enhanced the anti-inflammatory phenotype of the cells (Figure 2). However, exosomes are usually not available in sufficient quantities. It remains important to find other sources of exosomes. To address this problem, Si et al. (Chen et al., 2019; Liu et al., 2021b; Wei et al., 2021) attempted to utilize hASC which produces high amounts of exosomes. The transfection of hASC with lentivirus changed the content of miR-375 in cells, which in turn altered the amount of exosomal miR-375 secreted by cells. A large number of exosomes rich in miR-375 were obtained. After transfection with exosomes into hBMSCs, miR-375 inhibited the expression level of IGFBP3, a factor that negatively regulates osteogenesis, thus playing an osteogenic role in BMSCs. Yang et al. (2020) transfected BMSCs with exosomes rich in miR-130a-3p obtained from ADSC and found that miR-130a-3p targeted a variety of Wnt/β-catenin signaling pathway inhibitors, including DKK-1 and SIRT7.

Exosomes secreted by BMSCs also have the ability to promote bone regeneration and avoid the risk of genomic changes. Therefore, it is of great significance to explore the microRNA molecules contained therein and to transfect BMSCs to construct engineered cells (Hu et al., 2021; Huang et al., 2021). Zhang et al. (2020) used BMSC-derived exosomes with high expression of miR-22-3p to transfect the ovariectomized animal model with low expression of miR-22-3p and found that they targeted FTO and then inhibited the expression of the MYC gene, activating the PI3K/AKT signaling pathway and reversing the progression of osteoporosis.

For the application of exosomes, numerous studies have directly injected exosomes into the fracture site, or even into the abdominal cavity or intravenous injection (Hu et al., 2019; Zuo et al., 2019; Chen et al., 2020a; Zhang et al., 2021a; Zhang et al., 2021b; Duan et al., 2021; Hu et al., 2021; Nan et al., 2021; Wei et al., 2021; Xun et al., 2021). This method is not controllable, and the therapeutic effect is not reliable. Hence, the best method remains to construct engineered cells *in vitro* and transplant them *in vivo*.

#### 3.1.3 Engineered cells mediated by other organic materials

In addition to the viral vectors and exosomes mentioned earlier, plasmids are commonly used. Hu et al. (2018) transfected BMSCs with a plasmid carrying the miR-210-3p sequence and showed that miR-210-3p enhanced the osteogenic activity by targeting a variety of key osteogenic molecules, including HIF-1 and osteosclerosis protein. Furthermore, they planted plasmid-transfected BMSCs on PLLA and TCP scaffolds, respectively, demonstrating strong osteogenic activity in both non-weight-bearing and weight-bearing regions. Novel organic nanobiomaterials developed in recent years are regarded as reliable carriers, owing to their high transfection efficiency and cell innocuity. For example, Li et al. (2021a) reported a study using low molecular weight protamine (LMWP) rich in arginine as the carrier of the nucleic acid molecule miR-29b. However, due to the unstable structure of LMWP and low lysosomal escape that affects delivery efficiency, new stable organic carriers must be constructed.

### 3.2 miRNA-modified organic materials with the enhanced osteogenic ability

#### 3.2.1 miRNA-incorporated chitosan

CS has been applied in regenerative medicine, owing to its biocompatibility, antibacterial property, and drug-loading property (Balagangadharan et al., 2018; Yang et al., 2018). Balagangadharan et al. (2018) used CS as one of the main materials for the construction of the composite scaffold to carry the SMAD7-targeting antagonist miR-590-5p. The results showed that the composite scaffold could promote bone regeneration and angiogenesis. CS can be used to prepare hydrogels with a sustained-release function for the transfection of nucleic acids. Wei et al. (Xia et al., 2021) found that miR-328a-3p and miR-150-5p were highly expressed in cell vesicles secreted by injured neurons in the hippocampus in patients with traumatic brain injury. Extracellular exosomes were mixed with the ethylene glycol methacrylate chitosan (MeGC) hydrogel and then placed at the defect site, which was modified by fibronectin on the surface of vesicles to target osteoblast progenitors. Moreover, miR-328a-3p and miR-150-5p promote differentiation by targeting β-catenin suppressors FOXO4 and CBL, respectively. Sun et al. (2020b) prepared injectable gel materials by mixing miR-21 nanocapsules with o-carboxymethyl CS, which achieved the purpose of loading miR-21 and sustained release.

CS in combination with other materials helps enhance osteogenic properties. Jiang et al. (2020) used CS, sodium tripolyphosphate (TPP), and hyaluronic acid (HA) to prepare hydrogels carrying antagomiR-133a/b specific for targeting of miR-133a/b that silenced RUNX2, achieving the purpose of promoting osteogenic differentiation. In addition, it has been reported that miR-21/CS/HA-mixed gel coating also has the effect of promoting bone regeneration (Liu et al., 2017). These two studies showed that the combination of CS and HA could help improve the mechanical structure and stability of the CS hydrogel. As for natural organic biological materials, they have wide sources of materials and low cytotoxicity, but their transshipment capacity is limited and metabolism is difficult. Therefore, structural modification is needed to improve them.

#### 3.2.2 miRNA-incorporated polyethylene glycol

Organic polymers likewise attracted considerable attention in the development of nucleic acid carrier materials. PEG is widely studied in the field of biomaterial development, owing to its excellent softness and water solubility. As a high-quality hydrogel material, PEG can bind to miRNA in the hydrogel by self-assembly and has no adverse effect on the physicochemical properties of the PEG hydrogel (Nguyen et al., 2018). Gan et al. (2021) reported a cholesterol-modified miR-26a PEG hydrogel system. To achieve the purpose of controlled release, miR-26a was coupled to the PEG hydrogel by the UV-cutting ester bond. miR-26a promotes osteogenic differentiation of adipose stem cells by directly interfering with GSK3β synthesis. miR-26a further accelerates the progression of bone integration by inhibiting CTGF and thereby inhibiting RANKL-mediated osteoclast formation. Huynh et al. (Cong Truc et al., 2017) studied the PEG photodegradation hydrogel carrying a nucleic acid system, and a catalyst-free photodegradation hydrogel miR-20a delivery system was prepared using PEG-dipho-acrylate and eight-arm PEG-thiol. In the aforementioned studies, photodegradable PEG hydrogels modified with PEG were used, which demonstrates their excellent degradability and structural controllability as hydrogel materials. However, PEG itself degrades quickly and lacks pharmacokinetic stability such that it must be further modified or used in combination with other materials with a stable structure.

#### 3.2.3 miRNA-incorporated poly-lactic acid-glycolic acid

PLGA is a polymer of lactic and glycolic acids, and the polymerization of the two materials in different proportions achieves different degradation times. Therefore, there is a broad space for development in clinical applications. Qi et al. (2021) used PLGA to fabricate electrospun nanofibers for carrying miR-181. PLGA can be slowly degraded into lactic and glycolic acids *in vivo*, finally decomposed into CO_2_ and H_2_O, and release miR-181 encapsulated therein. MiR-181 targets mTORC1, a key molecule of the PTEN/PI3K/AKT signaling pathway, to promote bone regeneration in bone defects. The nanofibers constructed by this method have good softness and biocompatibility and exert a strong effect on cell aggregation and adhesion, which broadens the application prospect of PLGA.

**FIGURE 3:**
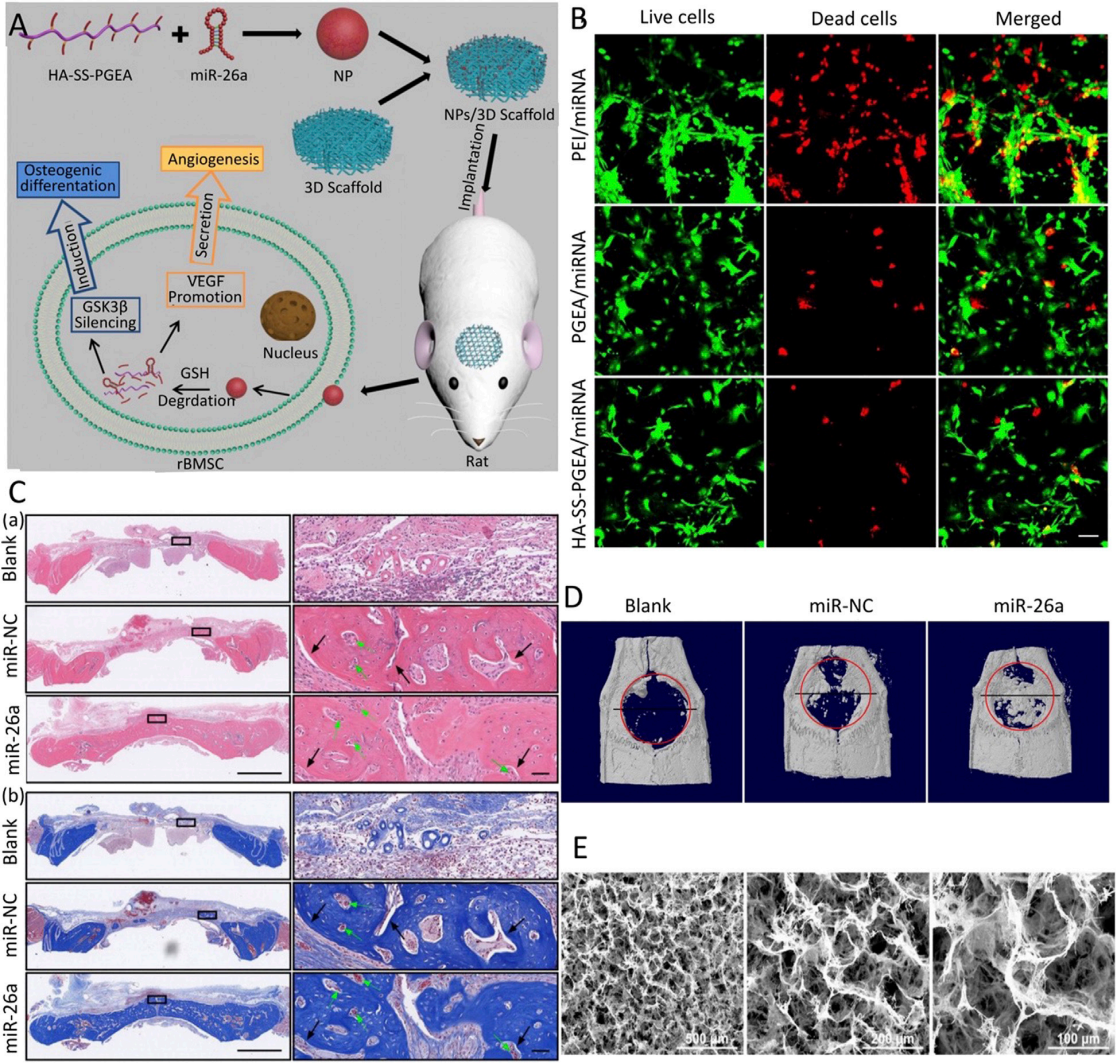
3D comb-shaped polycation (HA-SS-PGEA) nanofiber aerogel as miR-26a carrier for bone defect healing (Li et al., 2020). **(A)** Schematic diagram of the combination of HA-SS-PGEA and miR-26a nanoparticles (NPs) and the 3D nanofiber aerogel for bone defect repair. **(B)** CLSM images of live/dead staining mediated by various miRNA-containing NPs, including PEI, poly (glycidyl methacrylate) modified with target components, biodegradable groups, and short ethanolamine (PGEA), HA-SS-PGEA. **(C)** After 4 weeks of implantation, H&E staining **(A)** and Masson’s trichrome staining (MTC) **(B)** of rat cranial tissue were performed. **(D)** Representative planar X-ray films after 4 weeks of implantation. **(E)** SEM images of 3D mixed nanofiber aerogels. (blank, without 3D aerogel or NPs; miR-NC, negative control; CLSM, confocal laser scanning microscope; SEM, scanning electron microscope) Copyright 2020 Wiley-VCH GmbH. Reproduced with permission from John Wiley and Sons.

The combination of PLGA and PEG combines the advantages of both components to construct a degradable hydrogel. The composite hydrogel has the temperature-sensitive property of sol-gel-precipitation reversible conversion and prolongs the degradation time of PLGA (Yuan et al., 2019a). Lei et al. (2019) constructed the injectable temperature-controlled hydrogel using PLGA and PEG for the controlled release of monodisperse mesoporous silica microspheres loaded with miR-222. The defect area showed strong nerve and its innervated bone regeneration. Qian et al. (Li et al., 2021a) designed amphiphilic carriers by combining a hydrophobic PLGA segment with a hydrophilic PEG segment and then inserting PEI with the function of escape lysosome degradation and R9-G4-IKVAVW (RGI) peptide with the ability to recognize BMSCs and penetrate the cell membrane to form a new nucleic acid carrier for carrying miR-218 targeting the negative regulator of the Wnt/β-catenin signaling pathway. Li et al. (2020) constructed 3D nanofiber aerogels using PLGA-collagen-gelatin (PCG) and bioactive glass (BG) nanofibers, carrying a miR-26a delivery vector consisting of HA-disulfide-ethanolamine-functionalized poly (glycerol methacrylate). This hybrid carrier combines the advantages of various materials, has a bionic controllable porous structure and low biological toxicity, and is currently the appropriate nucleic acid transfection material (Figure 3). For PLGA materials, although they have a strong biological binding ability and structural controllability, the CO_2_ generated by their degradation will locally accumulate and cause an acidic microenvironment, which is hostile to bone regeneration. Therefore, PLGA materials must be further improved by combining with other materials that can eliminate the acidic microenvironment.

#### 3.2.4 miRNA-incorporated polymer polyethyleneimine

**FIGURE 4:**
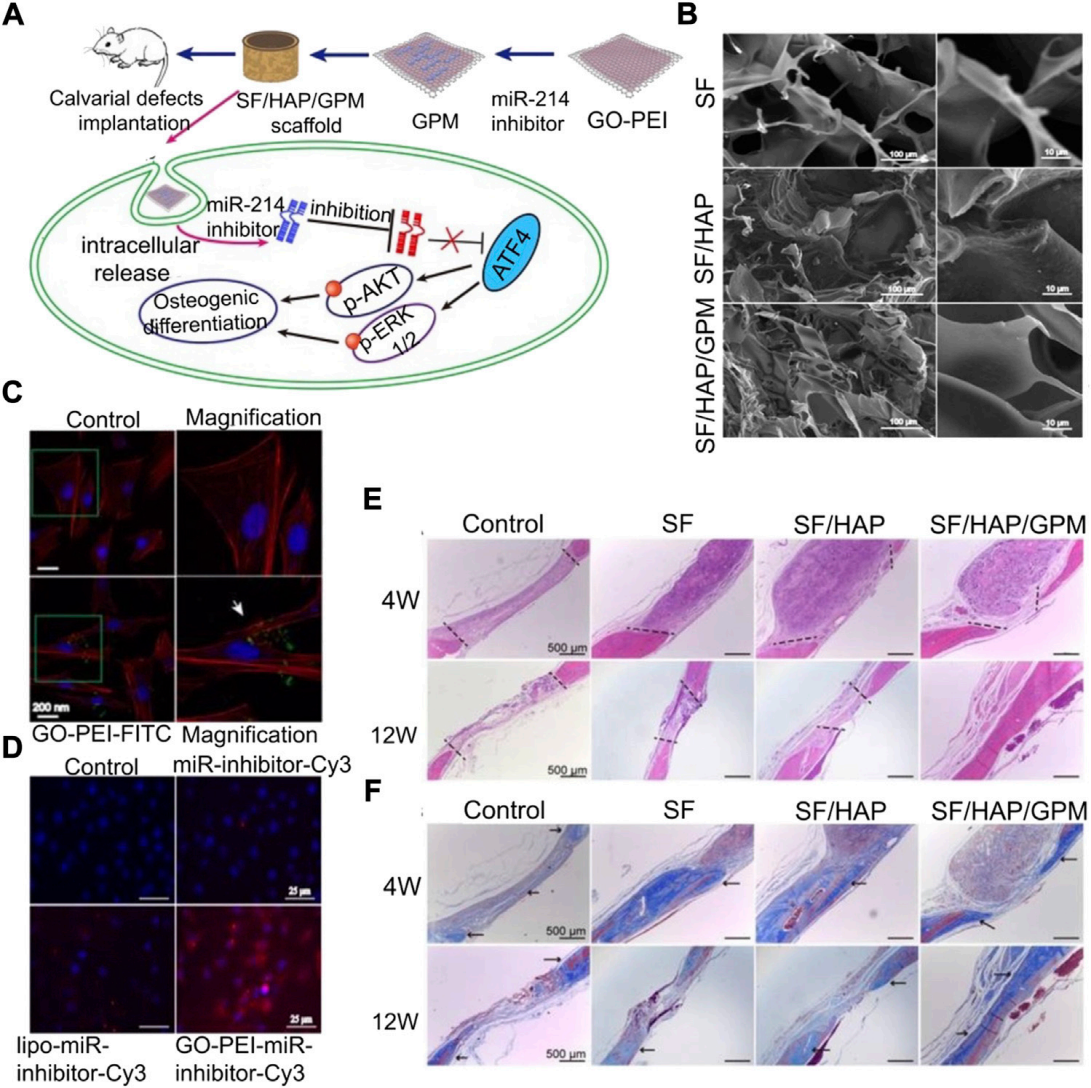
Silk fibroin/hydroxyapatite/graphene oxide–polyethyleneimine (SF/HAP/GO-PEI) scaffold carrying the miR-214 inhibitor promotes osteogenic differentiation (Ou et al., 2019). **(A)** Schematic diagram of the SF/HAP/GO-PEI scaffold carrying the miR-214 inhibitor to promote bone regeneration of bone defects. **(B)** SEM images of silk fibroin (SF), hybrid hydroxyapatite and SF scaffolds (SF/HAP), and SF/HAP/GO-PEI-miR-inhibitor (GPM). **(C)** CLSM fluorescence images showing non-GO-PEI-treated control and GO-PEI labeled with FITC (GO-PEI-FITC) in MC3T3-E1 cells. **(D)** Fluorescence images showing MC3T3-E1 cells treated with control, naked miR-inhibitor, Lipofectamine 2000, and GO-PEI after 24 h incubation. H&E staining **(E)** and MTC **(F)** were performed on the histological sections of the control group (defects without scaffolds served), SF, SF/HAP, and SF/HAP/GPM scaffolds after 4 and 12 weeks of implantation. Reproduced with permission from Ivyspring International Publisher.

PEI is a water-soluble polymer. As an miRNA-loaded material, PEI can enhance the ability to promote BMSC aggregation, adhesion, and osteogenic differentiation. However, the effect of PEI is not significant at low concentrations and has strong biotoxicity at high concentrations. It is, therefore, necessary to add other materials to overcome this limitation. Ou et al. (2019) combined negatively charged graphene oxide (GO) nanosheets with the cationic polymer PEI to the surface of hydroxyapatite (HAp), which could be used as an efficient non-toxic nucleic acid carrier. Moreover, PEI coating formed a porous shell on the surface of GO, which could be used as a slow-release system of the miR-214 inhibitor. miR-214 targets ATF4, and reducing the level of miR-214 in cells can activate PI3K/AKT and ERK1/2 signals and promote the deposition of calcium minerals (Figure 4). Bu et al. (2020) synthesized ascorbic acid-PEI carbon dots (CD) for carrying miR-2861 targeting HDAC5. Ascorbic acid is the raw material of CD, and it also has the potential to promote bone regeneration. CD has a small structure and active groups on the surface, but it will be absorbed by the lysosome. The lysosomal escape can be achieved by introducing PEI into the carrier, and the presence of CD can reduce the biological toxicity of PEI. Although PEI itself is highly toxic, its non-toxic treatment still has broad application prospects.

### 3.3 miRNA-modified inorganic biomaterials with enhanced osteogenic ability

#### 3.3.1 Metallic materials

Metallic materials have been widely used in the treatment of orthopedic diseases (Shuai et al., 2019). As the loading material of miRNA, metal materials can provide mechanical support in local bone defects. Different metal materials have their own unique characteristics, which can be used to construct different types of miRNA carriers. Common materials include titanium (Ti), aurum (Au), and zirconium (Zr) (Geng et al., 2020). Metal scaffolds, although they have good mechanical and antibacterial properties, they lack sufficient carrying capacity (Shuai et al., 2020). Long-term and stable nucleic acid carrying capacity is difficult to obtain by surface coating. Therefore, it is necessary to combine other materials to develop more efficient biological coatings or make metal scaffolds with electric charges to bind to miRNA through electrostatic adsorption (Yu et al., 2017; Li et al., 2021a).

**FIGURE 5:**
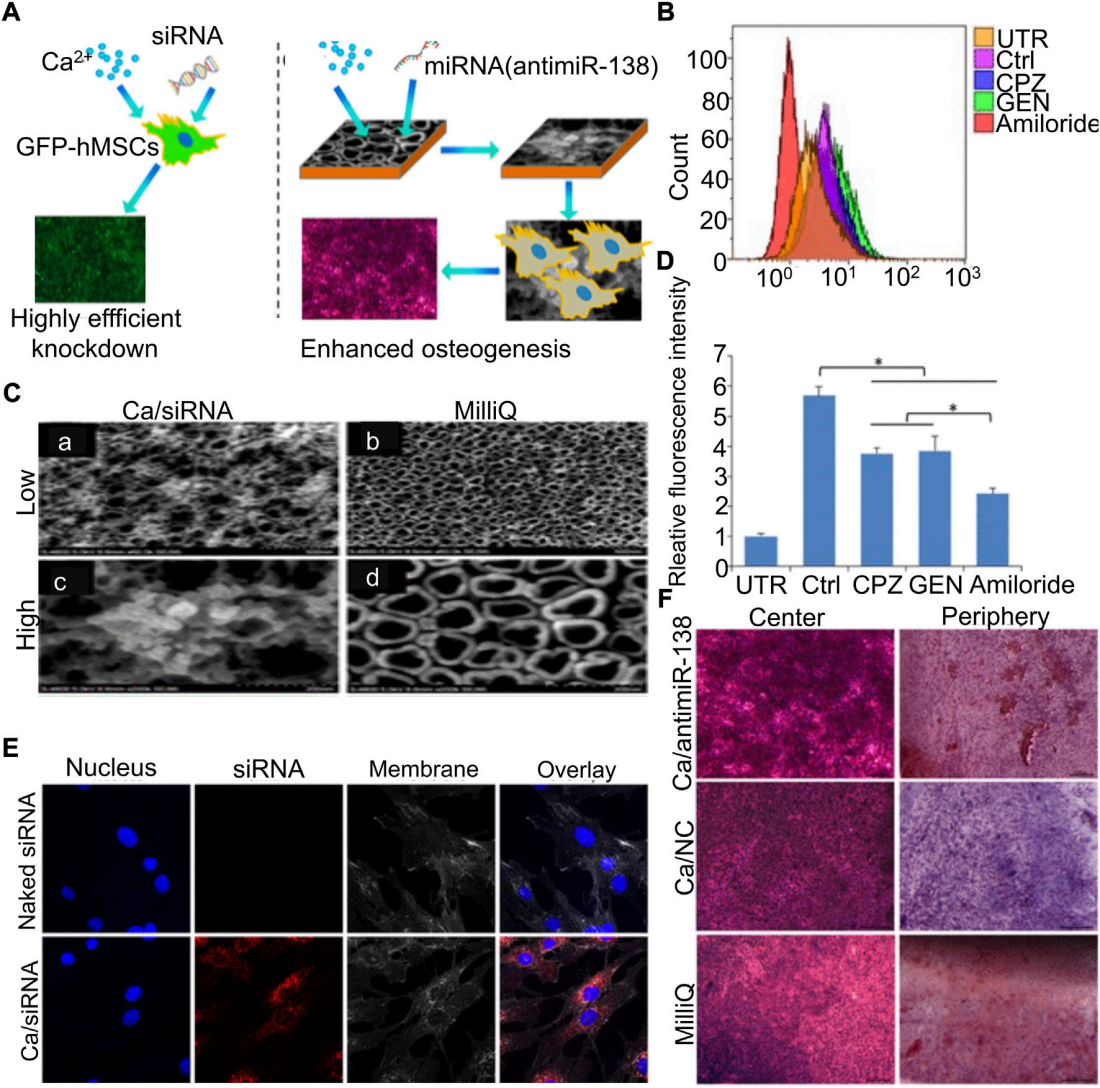
Calcium–microRNA complex combined with Ti scaffolds promoted osteogenic differentiation of hMSCs (Song et al., 2018). **(A)** Schematic diagram of the Ca/siRNA complex promoting osteogenic differentiation of BMSCs. Flow cytometry was used to analyze cell uptake **(B)** and quantification **(D)** after treatment with various cell phagocytosis inhibitors. **(C)** SEM observation of the TiO_2_ surface immersed in Ca/siRNA **(A,C)** or Milli-Q water **(B,D)**. **(E)** Cells were treated with naked siRNA and Ca/siRNA, respectively, and examined by CLSM. **(F)** hMSCs co-cultured with Ca/antimiR-138, Ca, and Milli-Q for 14 days, respectively, and stained with ARS. (UTR, untransfected cells; Ctrl, transfection without inhibitors; CPZ, chlorpromazine; GEN, genistein.) Copyright ^©^ 2018 American Chemical Society.

Ti metallic materials have been widely studied in the field of bone regeneration medicine, owing to their excellent load-bearing stability and biocompatibility (Sun et al., 2020b; Liu et al., 2021b). Liu et al. (2021b) isolated and obtained exosomes of BMSCs with high expression of miR-20a, which can target BAMBI competing with normal BMPs as the binding site of BMPR. These exosomes mixed with HA were injected into the pores of the porous Ti scaffold to provide local mechanical support and ensure the slow release of exosomes to improve osteogenesis. The surface modification or further development of metallic materials into new nanomaterials for carrying nucleic acids can play a better therapeutic role (Geng et al., 2020). Liu et al. (2017) first coupled antagomiR-204 with gold nanoparticles and dispersed them in PLGA solution, and then added Ti metal scaffolds. The implantation of composite scaffolds can achieve more efficient bone integration. Ca^2+^, as a divalent cation necessary for normal physiological activities of cells, can also combine anti-miR-138 to form cationic polymers under high concentration conditions. The loading of the Ca^2+^/anti-miR-138 complex into the TiO_2_ nanotube (NT) can form an amorphous uniform layer on the surface to achieve controlled release and mechanical support (Song et al., 2018) (Figure 5).

For miRNA delivery vectors prepared by Au, structural stability stably protects the structure and function of miRNA from opconditioning and RNase degradation. Abu-Laban et al. (2019) constructed Au nanoparticles and silver nanoparticles carrying miR-148b and miR-21, respectively, based on a chemical structure called the Diels–Alder reaction, and showed that at the corresponding light frequency, the nanometal would produce plasmon resonance. The heat shock forced the nucleic acid molecules out of the carrier to achieve stable release. Meng et al. (Yu et al., 2017) likewise focused on Au nanoparticles and developed ultra-small Au nanoparticles with a structure of less than 10 nm to make them easily dischargeable. Finally, PEI and liposome were used to wrap Au nanoparticles layer by layer to slowly release the loaded miR-5106. There are several biosafety issues for Au nanomaterials. Toxicity to cells and risk of immune inflammation may lead to local lytic destruction of bone defects. Future research must overcome the shortcomings while maintaining existing advantages.

Zr metal has excellent antibacterial properties and certain osteogenic properties, which can be used as a filler and loading material combined with miRNA to treat bone defects (Seweryn et al., 2020). Balagangadharan et al. (2018) used a nano-ZrO2/CS/nHAp metal–nonmetal hybrid scaffold loaded with miR-590-5p as a filling material for bone defects and showed that this composite material had excellent porosity and was helpful for bone and vascular regeneration. Currently, the application of Zr metal materials is still insufficient, and it is expected to be further developed in the future.

#### 3.3.2 Calcium orthophosphate

**FIGURE 6:**
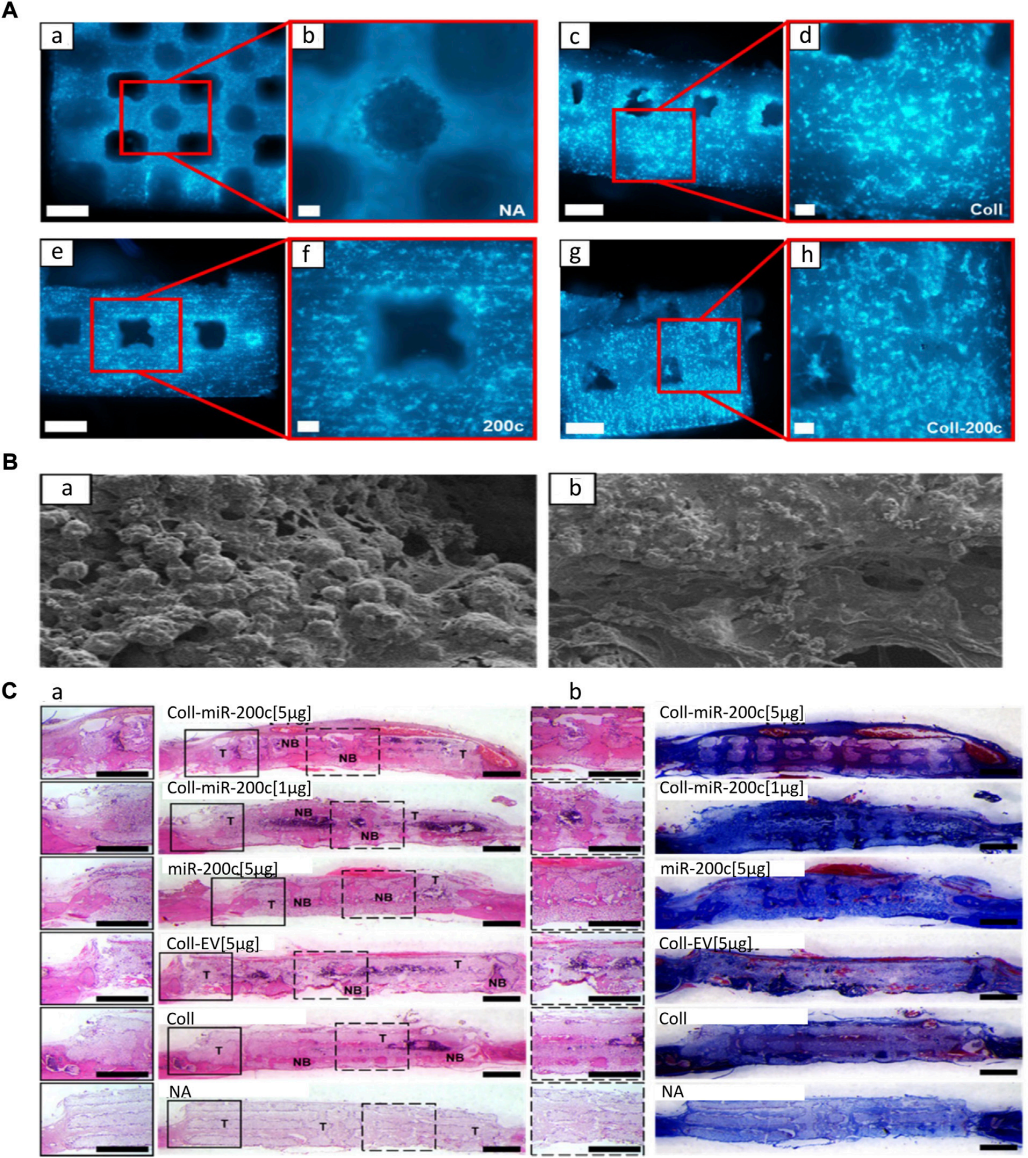
3D printing β-TCP scaffold loaded with miR-200c promotes bone regeneration (Remy et al., 2021). **(A)** DAPI nuclear staining image of hBMSCs on the β-TCP scaffold **(A–H)**. β-TCP scaffold without coating **(A,B)**; collagen (Coll)-coated β-TCP **(C,D)**; β-TCP containing miR-200c **(E,F)**; collagen coating, β-TCP **(G,H)** bound by miR-200c. Scales: 100-μm SEM images of the collagen network and matrix generated by 500 μm **(A,C,E,G)** and 100 μm **(B,D,F,H)**. **(B)** SEM images (100 μm) of the collagen network and matrix of hBMSCs attached to the surface of β-TCP scaffolds. **(C)** H&E **(A)** and MTC staining **(B)** of rat parietal bones 4 weeks after implantation. (Coll-miR-200c, collagen-coated scaffold containing miR-200c; EV, empty vector; NA, β-TCP scaffold alone.) Copyright ^©^ 2021 The Authors. Reproduced with permission from the American Chemical Society.

Calcium orthophosphate comprises the main material of artificial bone, and it is superior to other biomaterials in biocompatibility and osteogenesis. The implantation of calcium phosphate as the loading material of miRNA facilitates the growth of bone tissue and directly binds with new bone formation (Liu et al., 2021a). HAp, a type of calcium phosphate, has a similar structure to normal bone tissue, showing great potential for bone repair (Ou et al., 2019; Castano et al., 2020; Geng et al., 2020; Gan et al., 2021). However, HAp is brittle and cannot meet the mechanical support requirements of bone defects; hence, it must be combined with other materials to play a role in the field of regenerative medicine (Balagangadharan et al., 2018; Liu et al., 2021b). Moncal et al. (2019) prepared 3D printing scaffolds by melt extrusion with composite ink (PCL/PLGA/HAp) for loading the nano-silver-miR-148b hybrid carrier and adding type I collagen for filling and packaging. MiR-148b was mixed with silver nanoparticles to form thiol bonds and transfected into osteoblasts in a slow-release manner to directly target noggin in osteogenic differentiation, leading to transcriptional activation of osteogenic genes. β-TCP is a bioactive carrier similar to HAp, which has strong bone conductivity and is regarded as an ideal bone substitute (Hu et al., 2018; Remy et al., 2021). Remy et al. (2021) achieved a better bone growth effect by encapsulating miR-200c in type I collagen inner coating on 3D-printed porous β-TCP scaffolds (Figure 6). Although calcium orthophosphate scaffolds have good biocompatibility and osteogenic induction, their main disadvantage is mechanical brittleness. Therefore, it is necessary to evaluate and optimize the mechanical properties of miRNA-embedded calcium orthophosphate scaffolds in the study of bone defects in weight-bearing sites.

#### 3.3.3 Silicon-based materials

**FIGURE 7:**
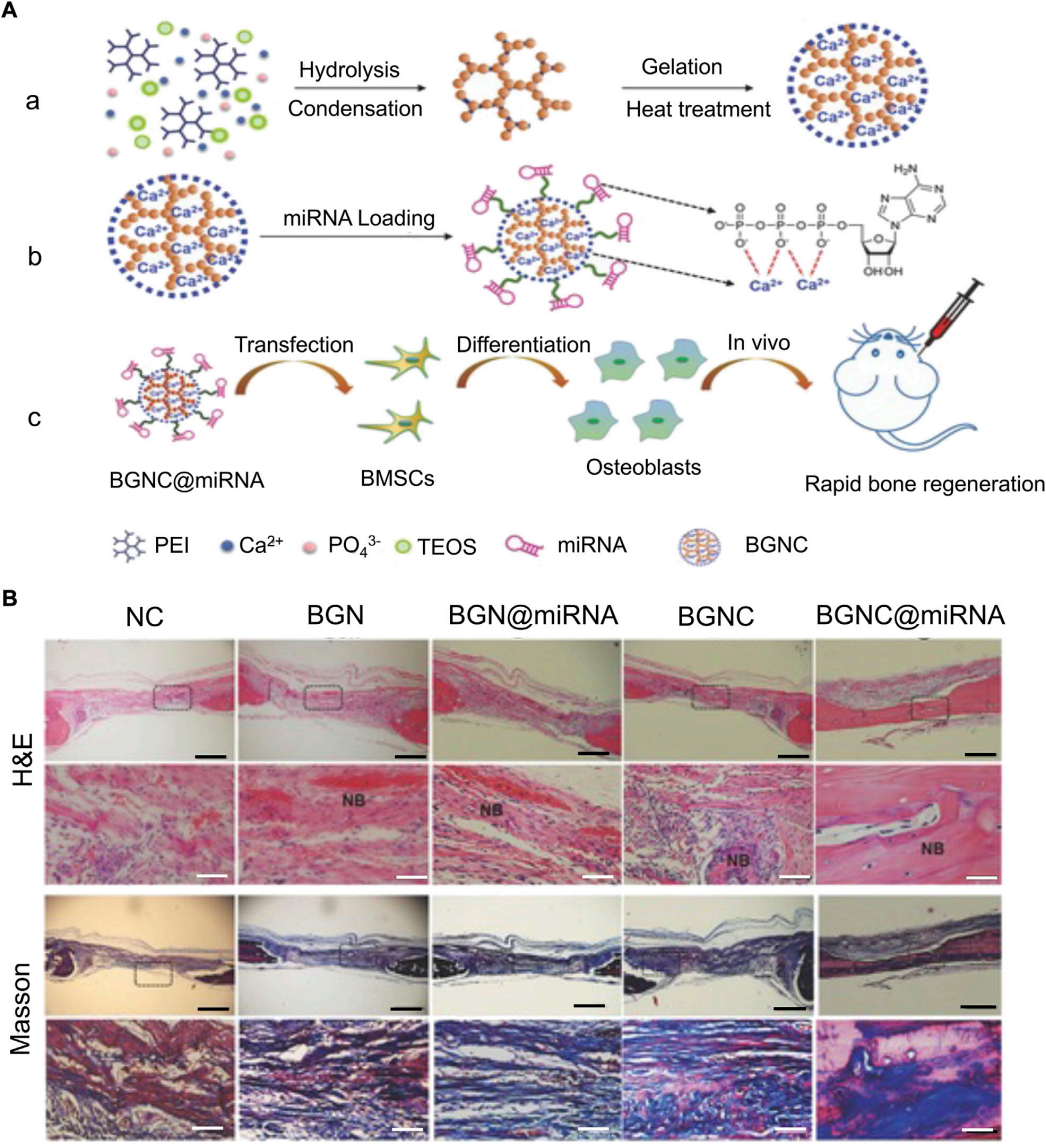
Monodispersed Bioactive Glass Nanoclusters (BGNCs) as vectors of miRNA promote osteogenic regeneration (Xue et al., 2017). **(A)** Schematic diagram of BGNC manufacturing and miRNA delivery applications. (a) Branched PEI-assisted synthesis of BGNCs. (b) miRNA loading. (c) miRNA delivery regulates the osteogenic activity of stem cells *in vitro* and bone regeneration *in vivo*. **(B)** Bone tissue samples were taken for H&E staining and MTC staining after 4 weeks of implantation in the rat parietal bones’ defect, with the black line representing 1 mm and the white line representing 100 µm. (NC, using the pure fibrin glue hydrogel as negative control; BGN, bioactive glass nanoparticle; BGN@miRNA, BGN with miRNA; BGNC@miRNA, BGNC with miRNA; TEOS, tetraethyl orthosilicate.) Copyright 2017 WILEY-VCH Verlag GmbH & Co. KGaA, Weinheim. Reproduced with permission from John Wiley and Sons.

Silicon-based materials have a good prospect in clinical application as they slowly degrade into non-toxic inorganic precursor silicic acid (Si(OH)_4_) *in vivo*, are carried by the blood and lymph system, and reach the kidney through the body fluid circulation to discharge *in vitro* (Lei et al., 2019). Therefore, miRNA carriers designed with degradation characteristics and non-toxicity of silicon-based materials have considerable advantages. Liu et al. (2021a) constructed dual-aperture calcium-silicon nanospheres for co-encapsulation of miR-210, angiogenesis gene drugs, and small-molecule osteogenic drug simvastatin to promote bone regeneration. Furthermore, monodisperse silica nanoparticles with a high surface area and controllable pores are suitable drug delivery systems. However, inorganic nanomaterials themselves lack sufficient nucleic acid-binding ability, which can be solved by combining them with organic materials. Yan et al. (2020) carried miR-26a with silica nanoparticles and covered it with PEI to prevent their degradation. Finally, miR-26a was successfully transferred into cells to promote osteogenic differentiation. Hosseinpour et al. (2021) reported that a lyophilized PEI-coated mesoporous silica nanoparticle was used as a sustained-release nucleic acid system to carry miR-26a-5p and promote osteogenic differentiation. Xue et al. (2017) constructed large-aperture monodisperse nano bioactive glass with PEI as the catalyst and template chain. Compared with traditional silica, this material has higher nucleic acid-carrying capacity (Figure 7). In addition to combining PEI to solve the defects of inorganic materials, it remains crucial to improve the ability of silicon-based materials to bind to miRNAs. Lei et al. (2019) used disulfide bonds and amino groups to modify MSN such that it had the ability to bind closely to miR-222 until the disulfide bonds were destroyed by the redox reaction of endogenous glutathione (GSH) after being swallowed by cells, and miR-222 was released. Silicon-based materials have reliable advantages as biomaterials, owing to their structural controllability, a wide range of sources, and biocompatibility. However, slow degradation limits their application prospects.

## 4 Conclusion and prospects

With the recent progress in gene research, the treatment of bone defects has been focused on nucleic acids. miRNA is a class of tiny non-coding RNAs that inhibit or degrade key molecules encoding osteogenic signaling pathways in mesenchymal stem cells by targeting mRNAs, or by interfering with the translation process. Ultimately, this changes the differentiation direction of mesenchymal stem cells, induces cell matrix calcification and precipitation, and realizes bone defect healing. We summarized miRNA-targeted key molecules in several well-studied intracellular signaling pathways, which provide a theoretical basis for the construction of biomaterials for clinical treatment. In addition, optimal nucleic acid transport and loading systems based on appropriate materials are essential to maintain effective nucleic acid transfection. Various studies showed that the construction of miRNA-engineered cells *in vitro* through viral or exosome vectors promotes osteogenic differentiation *in vitro* and bone repair *in vivo*. Furthermore, miRNA directly combines with organic materials, such as PEG and PLGA, and inorganic materials, such as metal, calcium phosphate, and silicon-based materials regulate the osteogenic differentiation of host cells.

Despite significant advances in miRNA load systems, numerous theoretical and technical challenges remain to be addressed. The cellular signaling pathway induced by miRNA in bone regeneration is not a comprehensive study. The role of the signaling pathway and its molecules is very complex, and it is necessary to further study key molecules in the signaling pathway to achieve a clear understanding of the osteogenic differentiation process of mesenchymal stem cells and provide ideas for clinical treatment and application. lncRNAs and circRNAs have spongy adsorption on miRNAs, which may interfere with its therapeutic effect. Therefore, future studies must further investigate the targeting relationship with miRNAs and the degree of influence at the level of lncRNAs and circRNAs, to more comprehensively evaluate its osteogenic ability. Current studies on the release kinetics of nucleic acid are insufficient, and further studies are required to determine the optimal mode of nucleic acid delivery so as to effectively utilize the metabolic regulation of miRNAs.

## References

[B1] Abu-Laban M. Hamal P. Arrizabalaga J. H. Forghani A. Dikkumbura A. S. Kumal R. R. (2019). Combinatorial delivery of miRNA-nanoparticle conjugates in human adipose stem cells for amplified osteogenesis. Small 15, 1902864. 10.1002/smll.201902864 PMC8530457 31725198

[B2] Akkouch A. Eliason S. Sweat M. E. Romero-Bustillos M. Zhu M. Qian F. (2019). Enhancement of MicroRNA-200c on osteogenic differentiation and bone regeneration by targeting sox2-mediated Wnt signaling and Klf4. Hum. Gene Ther. 30 (11), 1405–1418. 10.1089/hum.2019.019 31288577 PMC6854517

[B3] Arfat Y. Basra M. A. R. Shahzad M. Majeed K. Mahmood N. Munir H. (2018). miR-208a-3p suppresses osteoblast differentiation and inhibits bone formation by targeting ACVR1. Mol. Ther. - Nucleic Acids 11, 323–336. 10.1016/j.omtn.2017.11.009 29858067 PMC5992884

[B4] Bai H. Cui Y. Wang C. Wang Z. Luo W. Liu Y. (2020). 3D printed porous biomimetic composition sustained release zoledronate to promote osteointegration of osteoporotic defects. Mater. Des. 189, 108513. 10.1016/j.matdes.2020.108513

[B5] Balagangadharan K. Chandran S. V. Arumugam B. Saravanan S. Venkatasubbu G. D. Selvamurugan N. (2018). Chitosan/nano-hydroxyapatite/nano-zirconium dioxide scaffolds with miR-590-5p for bone regeneration. Int. J. Biol. Macromol. 111, 953–958. 10.1016/j.ijbiomac.2018.01.122 29415417

[B6] Brink O. (2021). The choice between allograft or demineralized bone matrix is not unambiguous in trauma surgery. Injury 52, S23–S28. 10.1016/j.injury.2020.11.013 33189329

[B7] Bu W. Xu X. Wang Z. Jin N. Liu L. Liu J. (2020). Ascorbic acid-PEI carbon dots with osteogenic effects as miR-2861 carriers to effectively enhance bone regeneration. ACS Appl. Mat. Interfaces 12 (45), 50287–50302. 10.1021/acsami.0c15425 33121247

[B8] Cao L. Liu W. Zhong Y. Zhang Y. Gao D. He T. (2020). Linc02349 promotes osteogenesis of human umbilical cord-derived stem cells by acting as a competing endogenous RNA for miR-25-3p and miR-33b-5p. Cell Prolif. 53 (5), e12814. 10.1111/cpr.12814 32346990 PMC7260076

[B9] Castano I. M. Raftery R. M. Chen G. Cavanagh B. Quinn B. Duffy G. R. (2020). Rapid bone repair with the recruitment of CD206(+)M2-like macrophages using non-viral scaffold-mediated miR-133a inhibition of host cells. Acta Biomater. 109, 267–279. 10.1016/j.actbio.2020.03.042 32251781

[B10] Chen S. Zheng Y. Zhang S. Jia L. Zhou Y. (2017). Promotion effects of miR-375 on the osteogenic differentiation of human adipose-derived mesenchymal stem cells. Stem Cell Rep. 8 (3), 773–786. 10.1016/j.stemcr.2017.01.028 PMC5355733 28262546

[B11] Chen S. Tang Y. Liu Y. Zhang P. Lv L. Zhang X. (2019). Exosomes derived from miR-375-overexpressing human adipose mesenchymal stem cells promote bone regeneration. Cell Prolif. 52 (5), e12669. 10.1111/cpr.12669 31380594 PMC6797519

[B12] Chen J. Liu M. Luo X. Peng L. Zhao Z. He C. (2020a). Exosomal miRNA-486-5p derived from rheumatoid arthritis fibroblast-like synoviocytes induces osteoblast differentiation through the Tob1/BMP/Smad pathway. Biomater. Sci. 8 (12), 3430–3442. 10.1039/c9bm01761e 32406432

[B13] Chen Z. Zhao F. Liang C. Hu L. Li D. Zhang Y. (2020b). Silencing of miR-138-5p sensitizes bone anabolic action to mechanical stimuli. Theranostics 10 (26), 12263–12278. 10.7150/thno.53009 33204341 PMC7667683

[B14] Chen Z. Zhang Y. Zhao F. Yin C. Yang C. Huai Y. (2021). miR-138-5p negatively regulates osteoblast differentiation through inhibiting beta-catenin under simulated microgravity in MC3T3-E1 cells. Acta Astronaut. 182, 240–250. 10.1016/j.actaastro.2021.01.052

[B15] Collon K. Gallo M. C. Lieberman J. R. (2021). Musculoskeletal tissue engineering: Regional gene therapy for bone repair. Biomaterials 275, 120901. 10.1016/j.biomaterials.2021.120901 34091300

[B16] Cong Truc H. Zheng Z. Minh Khanh N. McMillan A. Tonga G. Y. Rotello V. M. (2017). Cytocompatible catalyst-free photodegradable hydrogels for light-mediated RNA release to induce hMSC osteogenesis. ACS Biomater. Sci. Eng. 3 (9), 2011–2023. 10.1021/acsbiomaterials.6b00796 33440556 PMC13380960

[B17] Costa V. Carina V. Raimondi L. De Luca A. Bellavia D. Conigliaro A. (2019). MiR-33a controls hMSCS osteoblast commitment modulating the yap/taz expression through EGFR signaling regulation. Cells 8 (12), 1495. 10.3390/cells8121495 PMC6953103 31771093

[B18] Cui Q. Xing J. Yu M. Wang Y. Xu J. Gu Y. (2019a). Mmu-miR-185 depletion promotes osteogenic differentiation and suppresses bone loss in osteoporosis through the Bgn-mediated BMP/Smad pathway. Cell Death Dis. 10, 172. 10.1038/s41419-019-1428-1 30787286 PMC6382812

[B19] Cui Y. Zhu T. Li D. Li Z. Leng Y. Ji X. (2019b). Bisphosphonate-functionalized scaffolds for enhanced bone regeneration. Adv. Healthc. Mat. 8 (23), 1901073. 10.1002/adhm.201901073 31693315

[B20] Dai Z. Jin Y. Zheng J. Liu K. Zhao J. Zhang S. (2019). MiR-217 promotes cell proliferation and osteogenic differentiation of BMSCs by targeting DKK1 in steroid-associated osteonecrosis. Biomed. Pharmacother. 109, 1112–1119. 10.1016/j.biopha.2018.10.166 30551361

[B21] De Mattei M. Grassilli S. Pellati A. Brugnoli F. De Marchi E. Contartese D. (2020). Pulsed electromagnetic fields modulate miRNAs during osteogenic differentiation of bone mesenchymal stem cells: A possible role in the osteogenic-angiogenic coupling. Stem Cell Rev. Rep. 16 (5), 1005–1012. 10.1007/s12015-020-10009-6 32681233

[B22] Dong J. Xu X. Zhang Q. Yuan Z. Tan B. (2021). Critical implication of the PTEN/PI3K/AKT pathway during BMP2-induced heterotopic ossification. Mol. Med. Rep. 23 (4), 254. 10.3892/mmr.2021.11893 33537834 PMC7893754

[B23] Duan D.-Y. Tang J. Tian H.-T. Shi Y.-Y. Jia J. (2021). Adipocyte-secreted microvesicle-derived miR-148a regulates adipogenic and osteogenic differentiation by targeting Wnt5a/Ror2 pathway. Life Sci. 278, 119548. 10.1016/j.lfs.2021.119548 33930365

[B24] Feng L. Shi L. Lu Y.-f. Wang B. Tang T. Fu W.-m. (2018). Linc-ROR promotes osteogenic differentiation of mesenchymal stem cells by functioning as a competing endogenous RNA for miR-138 and miR-145. Mol. Ther. - Nucleic Acids 11, 345–353. 10.1016/j.omtn.2018.03.004 29858070 PMC5992460

[B25] Feng L. Zhang J.-F. Shi L. Yang Z.-M. Wu T.-Y. Wang H.-X. (2020). MicroRNA-378 suppressed osteogenesis of MSCs and impaired bone formation via inactivating wnt/β-catenin signaling. Mol. Ther. - Nucleic Acids 21, 1017–1028. 10.1016/j.omtn.2020.07.018 32829178 PMC7452050

[B26] Gan M. Zhou Q. Ge J. Zhao J. Wang Y. Yan Q. (2021). Precise *in-situ* release of microRNA from an injectable hydrogel induces bone regeneration. Acta biomater. 135, 289–303. 10.1016/j.actbio.2021.08.041 34474179

[B27] Geng Z. Yu Y. Li Z. Ma L. Zhu S. Liang Y. (2020). miR-21 promotes osseointegration and mineralization through enhancing both osteogenic and osteoclastic expression. Mater. Sci. Eng. C 111, 110785. 10.1016/j.msec.2020.110785 32279740

[B28] Godfrey T. C. Wildman B. J. Beloti M. M. Kemper A. G. Ferraz E. P. Roy B. (2018). The microRNA-23a cluster regulates the developmental HoxA cluster function during osteoblast differentiation. J. Biol. Chem. 293 (45), 17646–17660. 10.1074/jbc.RA118.003052 30242124 PMC6231122

[B29] Gundu S. Varshney N. Sahi A. K. Mahto S. K. (2022). Recent developments of biomaterial scaffolds and regenerative approaches for craniomaxillofacial bone tissue engineering. J. Polym. Res. 29 (3), 73. 10.1007/s10965-022-02928-4

[B30] Guo L. Chen K. Yuan J. Huang P. Xu X. Li C. (2019). Estrogen inhibits osteoclasts formation and bone resorption via microRNA-27a targeting PPAR gamma and APC. J. Cell. Physiol. 234 (1), 581–594. 10.1002/jcp.26788 30272823

[B31] Gyori D. Csete D. Benko S. Kulkarni S. Mandl P. Dobo-Nagy C. (2014). The phosphoinositide 3-kinase isoform PI3K beta regulates osteoclast-mediated bone resorption in humans and mice. Arthritis & Rheumatology 66 (8), 2210–2221. 10.1002/art.38660 24719382 PMC4314683

[B32] Hao Y. Lu C. Zhang B. Xu Z. Guo H. Zhang G. (2021). CircPVT1 up-regulation attenuates steroid-induced osteonecrosis of the femoral head through regulating miR-21-5p-mediated Smad7/TGF beta signalling pathway. J. Cell. Mol. Med. 25 (10), 4608–4622. 10.1111/jcmm.16294 33733589 PMC8107079

[B33] Hosseinpour S. Cao Y. Liu J. Xu C. Walsh L. J. (2021). Efficient transfection and long-term stability of rno-miRNA-26a-5p for osteogenic differentiation by large pore sized mesoporous silica nanoparticles. J. Mat. Chem. B 9 (9), 2275–2284. 10.1039/d0tb02756a 33606863

[B34] Hu Z. Wang Y. Sun Z. Wang H. Zhou H. Zhang L. (2015). miRNA-132-3p inhibits osteoblast differentiation by targeting Ep300 in simulated microgravity. Sci. Rep. 5, 18655. 10.1038/srep18655 26686902 PMC4685444

[B35] Hu B. Li Y. Wang M. Zhu Y. Zhou Y. Sui B. (2018). Functional reconstruction of critical-sized load-bearing bone defects using a Sclerostin-targeting miR-210-3p-based construct to enhance osteogenic activity. Acta Biomater. 76, 275–282. 10.1016/j.actbio.2018.06.017 29898419

[B36] Hu Y. Xu R. Chen C.-Y. Rao S.-S. Xia K. Huang J. (2019). Extracellular vesicles from human umbilical cord blood ameliorate bone loss in senile osteoporotic mice. Metabolism 95, 93–101. 10.1016/j.metabol.2019.01.009 30668962

[B37] Hu Z. Zhang L. Wang H. Wang Y. Tan Y. Dang L. (2020). Targeted silencing of miRNA-132-3p expression rescues disuse osteopenia by promoting mesenchymal stem cell osteogenic differentiation and osteogenesis in mice. Stem Cell Res. Ther. 11 (1), 58. 10.1186/s13287-020-1581-6 32054528 PMC7020585

[B38] Hu H. Wang D. Li L. Yin H. He G. Zhang Y. (2021). Role of microRNA-335 carried by bone marrow mesenchymal stem cells-derived extracellular vesicles in bone fracture recovery. Cell Death Dis. 12 (2), 156. 10.1038/s41419-021-03430-3 33542183 PMC7862274

[B39] Huang M. Li X. Zhou C. Si M. Zheng H. Chen L. (2020a). Noncoding RNA miR-205-5p mediates osteoporosis pathogenesis and osteoblast differentiation by regulating RUNX2. J. Cell. Biochem. 121 (10), 4196–4203. 10.1002/jcb.29599 31886577

[B40] Huang Y. Xiao D. Huang S. Zhuang J. Zheng X. Chang Y. (2020b). Circular RNA YAP1 attenuates osteoporosis through up-regulation of YAP1 and activation of Wnt/β-catenin pathway. Biomed. Pharmacother. 129, 110365. 10.1016/j.biopha.2020.110365 32768931

[B41] Huang Y. Xu Y. Feng S. He P. Sheng B. Ni J. (2021). miR-19b enhances osteogenic differentiation of mesenchymal stem cells and promotes fracture healing through the WWP1/Smurf2-mediated KLF5/β-catenin signaling pathway. Exp. Mol. Med. 53 (5), 973–985. 10.1038/s12276-021-00631-w 34035464 PMC8178348

[B42] Jia B. Wang Z. Sun X. Chen J. Zhao J. Qiu X. (2019). Long noncoding RNA LINC00707 sponges miR-370-3p to promote osteogenesis of human bone marrow-derived mesenchymal stem cells through upregulating WNT2B. Stem Cell Res. Ther. 10, 67. 10.1186/s13287-019-1161-9 30795799 PMC6387535

[B43] Jiang F. Yin F. Lin Y. Xia W. Zhou L. Pan C. (2020). The promotion of bone regeneration through CS/GP-CTH/antagomir-133a/b sustained release system. Nanomedicine Nanotechnol. Biol. Med. 24, 102116. 10.1016/j.nano.2019.102116 31672602

[B44] Jin H. Ji Y. Cui Y. Xu L. Liu H. Wang J. (2021). Simvastatin-incorporated drug delivery systems for bone regeneration. ACS Biomater. Sci. Eng. 7 (6), 2177–2191. 10.1021/acsbiomaterials.1c00462 33877804

[B45] Kaur S. Abu-Shahba A. G. Paananen R. O. Hongisto H. Hiidenmaa H. Skottman H. (2018). Small non-coding RNA landscape of extracellular vesicles from human stem cells. Sci. Rep. 8, 15503. 10.1038/s41598-018-33899-6 30341351 PMC6195565

[B46] Kim D.-K. Bandara G. Cho Y.-E. Komarow H. D. Donahue D. R. Karim B. (2021). Mastocytosis-derived extracellular vesicles deliver miR-23a and miR-30a into pre-osteoblasts and prevent osteoblastogenesis and bone formation. Nat. Commun. 12 (1), 2527. 10.1038/s41467-021-22754-4 33953168 PMC8100305

[B47] Kureel J. John A. A. Dixit M. Singh D. (2017). MicroRNA-467g inhibits new bone regeneration by targeting Ihh/Runx-2 signaling. Int. J. Biochem. Cell Biol. 85, 35–43. 10.1016/j.biocel.2017.01.018 28163186

[B48] Lei L. Liu Z. Yuan P. Jin R. Wang X. Jiang T. (2019). Injectable colloidal hydrogel with mesoporous silica nanoparticles for sustained co-release of microRNA-222 and aspirin to achieve innervated bone regeneration in rat mandibular defects. J. Mat. Chem. B 7 (16), 2722–2735. 10.1039/c9tb00025a 32255005

[B49] Li K.-C. Chang Y.-H. Hsu M.-N. Lo S.-C. Li W.-H. Hu Y.-C. (2017). Baculovirus-mediated miR-214 knockdown shifts osteoporotic ASCs differentiation and improves osteoporotic bone defects repair. Sci. Rep. 7, 16225. 10.1038/s41598-017-16547-3 29176755 PMC5701180

[B50] Li H. Fan J. Fan L. Li T. Yang Y. Xu H. (2018). MiRNA-10b reciprocally stimulates osteogenesis and inhibits adipogenesis partly through the TGF-β/SMAD2 signaling pathway. Aging Dis. 9 (6), 1058–1073. 10.14336/ad.2018.0214 30574418 PMC6284771

[B51] Li R. Wang H. John J. V. Song H. Teusink M. J. Xie J. (2020). 3D hybrid nanofiber aerogels combining with nanoparticles made of a biocleavable and targeting polycation and MiR-26a for bone repair. Adv. Funct. Mat. 30 (49), 2005531. 10.1002/adfm.202005531 PMC8315031 34326714

[B52] Li Q. Hu Z. Rong X. Chang B. Liu X. (2021a). Multifunctional polyplex micelles for efficient microRNA delivery and accelerated osteogenesis. Nanoscale 13 (28), 12198–12211. 10.1039/d1nr02638k 34231613 PMC10041663

[B53] Li S. Liu Y. Tian T. Zhang T. Lin S. Zhou M. (2021b). Bioswitchable delivery of microRNA by framework nucleic acids: Application to bone regeneration. Small 17, 2104359. 10.1002/smll.202104359 34716653

[B54] Lian W.-S. Ko J.-Y. Chen Y.-S. Ke H.-J. Hsieh C.-K. Kuo C.-W. (2019). MicroRNA-29a represses osteoclast formation and protects against osteoporosis by regulating PCAF-mediated RANKL and CXCL12. Cell Death Dis. 10, 705. 10.1038/s41419-019-1942-1 31543513 PMC6755134

[B55] Lin Z. He H. Wang M. Liang J. (2019). MicroRNA-130a controls bone marrow mesenchymal stem cell differentiation towards the osteoblastic and adipogenic fate. Cell Prolif. 52 (6), e12688. 10.1111/cpr.12688 31557368 PMC6869834

[B56] Liu J. Wang H. Zuo Y. Farmer S. R. (2006). Functional interaction between peroxisome proliferator-activated receptor gamma and beta-catenin. Mol. Cell. Biol. 26 (15), 5827–5837. 10.1128/mcb.00441-06 16847334 PMC1592783

[B57] Liu X. Tan N. Zhou Y. Wei H. Ren S. Yu F. (2017). Delivery of antagomiR204-conjugated gold nanoparticles from PLGA sheets and its implication in promoting osseointegration of titanium implant in type 2 diabetes mellitus. Int. J. Nanomedicine 12, 7089–7101. 10.2147/ijn.S124584 29026303 PMC5627761

[B58] Liu H. Dong Y. Feng X. Li L. Jiao Y. Bai S. (2019). miR-34a promotes bone regeneration in irradiated bone defects by enhancing osteoblastic differentiation of mesenchymal stromal cells in rats. Stem Cell Res. Ther. 10, 180. 10.1186/s13287-019-1285-y 31215466 PMC6582588

[B59] Liu J. Cui Y. Kuang Y. Xu S. Lu Q. Diao J. (2021a). Hierarchically porous calcium-silicon nanosphere-enabled co-delivery of microRNA-210 and simvastatin for bone regeneration. J. Mat. Chem. B 9 (16), 3573–3583. 10.1039/d1tb00063b 33909742

[B60] Liu W. Huang J. Chen F. Xie D. Wang L. Ye C. (2021b). MSC-derived small extracellular vesicles overexpressing miR-20a promoted the osteointegration of porous titanium alloy by enhancing osteogenesis via targeting BAMBI. Stem Cell Res. Ther. 12 (1), 348. 10.1186/s13287-021-02303-y 34134765 PMC8207591

[B61] Long H. Zhu Y. Lin Z. Wan J. Cheng L. Zeng M. (2019). miR-381 modulates human bone mesenchymal stromal cells (BMSCs) osteogenesis via suppressing Wnt signaling pathway during atrophic nonunion development. Cell Death Dis. 10, 470. 10.1038/s41419-019-1693-z 31209205 PMC6572824

[B62] Lu X.-D. Han W.-X. Liu Y.-X. (2019). Suppression of miR-451a accelerates osteogenic differentiation and inhibits bone loss via Bmp6 signaling during osteoporosis. Biomed. Pharmacother. 120, 109378. 10.1016/j.biopha.2019.109378 31541885

[B63] Luo Y. Cao X. Chen J. Gu J. Zhao J. Sun J. (2018). MicroRNA-224 suppresses osteoblast differentiation by inhibiting SMAD4. J. Cell. Physiol. 233 (10), 6929–6937. 10.1002/jcp.26596 29693254

[B64] Luo Y. Ge R. Wu H. Ding X. Song H. Ji H. (2019). The osteogenic differentiation of human adipose-derived stem cells is regulated through the let-7i-3p/LEF1/β-catenin axis under cyclic strain. Stem Cell Res. Ther. 10 (1), 339. 10.1186/s13287-019-1470-z 31753039 PMC6873506

[B65] Luo Y. Ding X. Ji H. Li M. Song H. Li S. (2020). MicroRNA-503-3p affects osteogenic differentiation of human adipose-derived stem cells by regulation of Wnt2 and Wnt7b under cyclic strain. Stem Cell Res. Ther. 11 (1), 318. 10.1186/s13287-020-01842-0 32711579 PMC7382842

[B66] Ma X. Bian Y. Yuan H. Chen N. Pan Y. Zhou W. (2020a). Human amnion-derived mesenchymal stem cells promote osteogenic differentiation of human bone marrow mesenchymal stem cells via H19/miR-675/APC axis. Aging 12 (11), 10527–10543. 10.18632/aging.103277 32434960 PMC7346082

[B67] Ma X. Fan C. Wang Y. Du Y. Zhu Y. Liu H. (2020b). MiR-137 knockdown promotes the osteogenic differentiation of human adipose-derived stem cells via the LSD1/BMP2/SMAD4 signaling network. J. Cell. Physiol. 235 (2), 909–919. 10.1002/jcp.29006 31241766

[B68] Makitie R. E. Hackl M. Niinimaki R. Kakko S. Grillari J. Makitie O. (2018). Altered MicroRNA profile in osteoporosis caused by impaired WNT signaling. J. Clin. Endocrinol. Metab. 103 (5), 1985–1996. 10.1210/jc.2017-02585 29506076

[B69] Marycz K. Smieszek A. Marcinkowska K. Sikora M. Turlej E. Sobierajska P. (2021). Nanohydroxyapatite (nHAp) doped with iron oxide nanoparticles (IO), miR-21 and miR-124 under magnetic field conditions modulates osteoblast viability, reduces inflammation and inhibits the growth of osteoclast - a novel concept for osteoporosis treatment: Part 1. Int. J. Nanomedicine 16, 3429–3456. 10.2147/ijn.S303412 34040372 PMC8140937

[B70] Mi B. Xiong Y. Yan C. Chen L. Xue H. Panayi A. C. (2020). Methyltransferase-like 3-mediated N6-methyladenosine modification of miR-7212-5p drives osteoblast differentiation and fracture healing. J. Cell. Mol. Med. 24 (11), 6385–6396. 10.1111/jcmm.15284 32307908 PMC7294157

[B71] Moncal K. K. Aydin R. S. T. Abu-Laban M. Heo D. N. Rizk E. Tucker S. M. (2019). Collagen-infilled 3D printed scaffolds loaded with miR-148b-transfected bone marrow stem cells improve calvarial bone regeneration in rats. Mater. Sci. Eng. C 105, 110128. 10.1016/j.msec.2019.110128 PMC6761997 31546389

[B72] Nan K. Zhang Y. Zhang X. Li D. Zhao Y. Jing Z. (2021). Exosomes from miRNA-378-modified adipose-derived stem cells prevent glucocorticoid-induced osteonecrosis of the femoral head by enhancing angiogenesis and osteogenesis via targeting miR-378 negatively regulated suppressor of fused (Sufu). Stem Cell Res. Ther. 12 (1), 331. 10.1186/s13287-021-02390-x 34099038 PMC8186190

[B73] Nguyen M. K. Jeon O. Dang P. N. Huynh C. T. Varghai D. Riazi H. (2018). RNA interfering molecule delivery from *in situ* forming biodegradable hydrogels for enhancement of bone formation in rat calvarial bone defects. Acta Biomater. 75, 105–114. 10.1016/j.actbio.2018.06.007 29885529 PMC6119505

[B74] Ou L. Lan Y. Feng Z. Feng L. Yang J. Liu Y. (2019). Functionalization of SF/HAP scaffold with GO-PEI-miRNA inhibitor complexes to enhance bone regeneration through activating transcription factor 4. Theranostics 9 (15), 4525–4541. 10.7150/thno.34676 31285777 PMC6599658

[B75] Peng W. Zhu S. Chen J. Wang J. Rong Q. Chen S. (2019). Hsa_circRNA_33287 promotes the osteogenic differentiation of maxillary sinus membrane stem cells via miR-214-3p/Runx3. Biomed. Pharmacother. 109, 1709–1717. 10.1016/j.biopha.2018.10.159 30551425

[B76] Qi P. Niu Y. Wang B. (2021). MicroRNA-181a/b-1-encapsulated PEG/PLGA nanofibrous scaffold promotes osteogenesis of human mesenchymal stem cells. J. Cell. Mol. Med. 25 (12), 5744–5752. 10.1111/jcmm.16595 33991050 PMC8184675

[B77] Remy M. T. Akkouch A. He L. Eliason S. Sweat M. E. Krongbaramee T. (2021). Rat calvarial bone regeneration by 3D-printed beta-tricalcium phosphate incorporating MicroRNA-200c. ACS Biomater. Sci. Eng. 7 (9), 4521–4534. 10.1021/acsbiomaterials.0c01756 34437807 PMC8441974

[B78] Seweryn A. Pielok A. Lawniczak-Jablonska K. Pietruszka R. Marcinkowska K. Sikora M. (2020). Zirconium oxide thin films obtained by atomic layer deposition Technology abolish the anti-osteogenic effect resulting from miR-21 inhibition in the pre-osteoblastic MC3T3 cell line. Int. J. Nanomedicine 15, 1595–1610. 10.2147/ijn.S237898 32210554 PMC7069564

[B79] Shen W. Sun B. Zhou C. Ming W. Zhang S. Wu X. (2020). CircFOXP1/FOXP1 promotes osteogenic differentiation in adipose-derived mesenchymal stem cells and bone regeneration in osteoporosis via miR-33a-5p. J. Cell. Mol. Med. 24 (21), 12513–12524. 10.1111/jcmm.15792 32996692 PMC7687013

[B80] Shi Z.-l. Zhang H. Fan Z.-y. Ma W. Song Y.-z. Li M. (2020). Long noncoding RNA LINC00314 facilitates osteogenic differentiation of adipose-derived stem cells through the hsa-miR-129-5p/GRM5 axis via the Wnt signaling pathway. Stem Cell Res. Ther. 11 (1), 240. 10.1186/s13287-020-01754-z 32552820 PMC7302136

[B81] Shuai C. J. Xu Y. Feng P. Wang G. Y. Xiong S. X. Peng S. P. (2019). Antibacterial polymer scaffold based on mesoporous bioactive glass loaded with *in situ* grown silver. Chem. Eng. J. 374, 304–315. 10.1016/j.cej.2019.03.273

[B82] Shuai C. J. Liu G. F. Yang Y. W. Qi F. W. Peng S. P. Yang W. J. (2020). A strawberry-like Ag-decorated barium titanate enhances piezoelectric and antibacterial activities of polymer scaffold. Nano Energy 74, 104825. 10.1016/j.nanoen.2020.104825

[B83] Smieszek A. Marcinkowska K. Pielok A. Sikora M. Valihrach L. Marycz K. (2020). The role of miR-21 in osteoblasts-osteoclasts coupling *in vitro* . Cells 9 (2), 479. 10.3390/cells9020479 PMC7072787 32093031

[B84] Song W. Yang C. Le D. Q. S. Zhang Y. Kjems J. (2018). Calcium-MicroRNA complex-functionalized nanotubular implant surface for highly efficient transfection and enhanced osteogenesis of mesenchymal stem cells. ACS Appl. Mat. Interfaces 10 (9), 7756–7764. 10.1021/acsami.7b18289 29420881

[B85] Soszyńska A. Klimczewska K. Suwińska A. (2019). FGF/ERK signaling pathway: How it operates in mammalian preimplantation embryos and embryo-derived stem cells. Int. J. Dev. Biol. 63 (3-4-5), 171–186. 10.1387/ijdb.180408as 31058295

[B86] Srinaath N. Balagangadharan K. Pooja V. Paarkavi U. Trishla A. Selvamurugan N. (2019). Osteogenic potential of zingerone, a phenolic compound in mouse mesenchymal stem cells. Biofactors 45 (4), 575–582. 10.1002/biof.1515 31091349

[B87] Sun M. Hu L. Wang S. Huang T. Zhang M. Yang M. (2020a). Circulating MicroRNA-19b identified from osteoporotic vertebral compression fracture patients increases bone formation. J. Bone Min. Res. 35 (2), 306–316. 10.1002/jbmr.3892 31614022

[B88] Sun X. Li X. Qi H. Hou X. Zhao J. Yuan X. (2020b). MiR-21 nanocapsules promote early bone repair of osteoporotic fractures by stimulating the osteogenic differentiation of bone marrow mesenchymal stem cells. J. Orthop. Transl. 24, 76–87. 10.1016/j.jot.2020.04.007 PMC7349941 32695607

[B89] Tang Z. Xu T. Li Y. Fei W. Yang G. Hong Y. (2020). Inhibition of CRY2 by STAT3/miRNA-7-5p promotes osteoblast differentiation through upregulation of CLOCK/BMAL1/P300 expression. Mol. Ther. - Nucleic Acids 19, 865–876. 10.1016/j.omtn.2019.12.020 31982773 PMC6994415

[B90] Vishal M. Vimalraj S. Ajeetha R. Gokulnath M. Keerthana R. He Z. (2017). MicroRNA-590-5p stabilizes Runx2 by targeting Smad7 during osteoblast differentiation. J. Cell. Physiol. 232 (2), 371–380. 10.1002/jcp.25434 27192628

[B91] Wang W. Yang L. Zhang D. Gao C. Wu J. Zhu Y. (2018a). MicroRNA-218 negatively regulates osteoclastogenic differentiation by repressing the nuclear factor-kappa B signaling pathway and targeting tumor necrosis factor receptor 1. Cell. Physiol. biochem. 48 (1), 339–347. 10.1159/000491740 30016774

[B92] Wang Y. Liu W. Liu Y. Cui J. Zhao Z. Cao H. (2018b). Long noncoding RNA H19 mediates LCoR to impact the osteogenic and adipogenic differentiation of mBMSCs in mice through sponging miR-188. J. Cell. Physiol. 233 (9), 7435–7446. 10.1002/jcp.26589 29663375 PMC13482115

[B93] Wang C.-G. Hu Y.-H. Su S.-L. Zhong D. (2020a). LncRNA DANCR and miR-320a suppressed osteogenic differentiation in osteoporosis by directly inhibiting the Wnt/β-catenin signaling pathway. Exp. Mol. Med. 52 (8), 1310–1325. 10.1038/s12276-020-0475-0 32778797 PMC8080634

[B94] Wang R. Zhang H. Ding W. Fan Z. Ji B. Ding C. (2020b). miR-143 promotes angiogenesis and osteoblast differentiation by targeting HDAC7. Cell Death Dis. 11 (3), 179. 10.1038/s41419-020-2377-4 32152265 PMC7062786

[B95] Wang T. Zhang C. Wu C. Liu J. Yu H. Zhou X. (2020c). miR-765 inhibits the osteogenic differentiation of human bone marrow mesenchymal stem cells by targeting BMP6 via regulating the BMP6/Smad1/5/9 signaling pathway. Stem Cell Res. Ther. 11 (1), 62. 10.1186/s13287-020-1579-0 32059748 PMC7023766

[B96] Wei Y. Ma H. Zhou H. Yin H. Yang J. Song Y. (2021). miR-424-5p shuttled by bone marrow stem cells-derived exosomes attenuates osteogenesis via regulating WIF1-mediated Wnt/β-catenin axis. Aging 13 (13), 17190–17201. 10.18632/aging.203169 34229300 PMC8312462

[B97] Wu R.-W. Lian W.-S. Chen Y.-S. Ko J.-Y. Wang S.-Y. Jahr H. (2021). Piezoelectric microvibration mitigates estrogen loss-induced osteoporosis and promotes Piezo1, MicroRNA-29a, and Wnt3a signaling in osteoblasts. Int. J. Mol. Sci. 22 (17), 9476. 10.3390/ijms22179476 34502380 PMC8431199

[B98] Xia W. Xie J. Cai Z. Liu X. Wen J. Cui Z.-K. (2021). Damaged brain accelerates bone healing by releasing small extracellular vesicles that target osteoprogenitors. Nat. Commun. 12 (1), 6043. 10.1038/s41467-021-26302-y 34654817 PMC8519911

[B99] Xiao J. Qin S. Li W. Yao L. Huang P. Liao J. (2020). Osteogenic differentiation of rat bone mesenchymal stem cells modulated by MiR-186 via SIRT6. Life Sci. 253, 117660. 10.1016/j.lfs.2020.117660 32294474

[B100] Xiong Y. Cao F. Hu L. Yan C. Chen L. Panayi A. C. (2019). miRNA-26a-5p accelerates healing via downregulation of PTEN in fracture patients with traumatic brain injury. Mol. Ther. - Nucleic Acids 17, 223–234. 10.1016/j.omtn.2019.06.001 31272072 PMC6610686

[B101] Xiong A. He Y. Gao L. Li G. Weng J. Kang B. (2020a). Smurf1-targeting miR-19b-3p-modified BMSCs combined PLLA composite scaffold to enhance osteogenic activity and treat critical-sized bone defects. Biomater. Sci. 8 (21), 6069–6081. 10.1039/d0bm01251c 33000773

[B102] Xiong Y. Chen L. Yan C. Endo Y. Mi B. Liu G. (2020b). The lncRNA rhno1/miR-6979-5p/BMP2 Axis modulates osteoblast differentiation. Int. J. Biol. Sci. 16 (9), 1604–1615. 10.7150/ijbs.38930 32226305 PMC7097916

[B103] Xiong Y. Chen L. Yan C. Zhou W. Yu T. Sun Y. (2020c). M2 Macrophagy-derived exosomal miRNA-5106 induces bone mesenchymal stem cells towards osteoblastic fate by targeting salt-inducible kinase 2 and 3. J. Nanobiotechnology 18 (1), 66. 10.1186/s12951-020-00622-5 32345321 PMC7189726

[B104] Xu T. Luo Y. Wang J. Zhang N. Gu C. Li L. (2020). Exosomal miRNA-128-3p from mesenchymal stem cells of aged rats regulates osteogenesis and bone fracture healing by targeting Smad5. J. Nanobiotechnology 18 (1), 47. 10.1186/s12951-020-00601-w 32178675 PMC7077029

[B105] Xue Y. Guo Y. Yu M. Wang M. Ma P. X. Lei B. (2017). Monodispersed bioactive glass Nanoclusters with ultralarge pores and intrinsic exceptionally high miRNA loading for efficiently enhancing bone regeneration. Adv. Healthc. Mat. 6 (20), 1700630. 10.1002/adhm.201700630 28737023

[B106] Xun J. Li C. Liu M. Mei Y. Zhou Q. Wu B. (2021). Serum exosomes from young rats improve the reduced osteogenic differentiation of BMSCs in aged rats with osteoporosis after fatigue loading *in vivo* . Stem Cell Res. Ther. 12 (1), 424. 10.1186/s13287-021-02449-9 34315544 PMC8314589

[B107] Yan J. Lu X. Zhu X. Hu X. Wang L. Qian J. (2020). Effects of miR-26a on osteogenic differentiation of bone marrow mesenchymal stem cells by a mesoporous silica nanoparticle - PEI - peptide system. Int. J. Nanomedicine 15, 497–511. 10.2147/ijn.S228797 32158207 PMC6986258

[B108] Yang Y. Chu L. Yang S. Zhang H. Qin L. Guillaume O. (2018). Dual-functional 3D-printed composite scaffold for inhibiting bacterial infection and promoting bone regeneration in infected bone defect models. Acta Biomater. 79, 265–275. 10.1016/j.actbio.2018.08.015 30125670

[B109] Yang C. Liu X. Zhao K. Zhu Y. Hu B. Zhou Y. (2019). miRNA-21 promotes osteogenesis via the PTEN/PI3K/Akt/HIF-1 pathway and enhances bone regeneration in critical size defects. Stem Cell Res. Ther. 10, 65. 10.1186/s13287-019-1168-2 30795815 PMC6387542

[B110] Yang S. Guo S. Tong S. Sun X. (2020). Exosomal miR‐130a‐3p regulates osteogenic differentiation of Human Adipose‐Derived stem cells through mediating SIRT7/Wnt/β‐catenin axis. Cell Prolif. 53 (10), e12890. 10.1111/cpr.12890 32808361 PMC7574877

[B111] Yin Q. Wang J. Fu Q. Gu S. Rui Y. (2018). CircRUNX2 through has-miR-203 regulates RUNX2 to prevent osteoporosis. J. Cell. Mol. Med. 22 (12), 6112–6121. 10.1111/jcmm.13888 30324718 PMC6237596

[B112] You Y. Ma W. Wang F. a. Jiao G. Zhang L. Zhou H. (2021). Ortho-silicic acid enhances osteogenesis of osteoblasts through the upregulation of miR-130b which directly targets PTEN. Life Sci. 264, 118680. 10.1016/j.lfs.2020.118680 33130075

[B113] Yu M. Lei B. Gao C. Yan J. Ma P. X. (2017). Optimizing surface-engineered ultra-small gold nanoparticles for highly efficient miRNA delivery to enhance osteogenic differentiation of bone mesenchymal stromal cells. Nano Res. 10 (1), 49–63. 10.1007/s12274-016-1265-9

[B114] Yu T. You X. Zhou H. He W. Li Z. Li B. (2020). MiR-16-5p regulates postmenopausal osteoporosis by directly targeting VEGFA. Aging 12 (10), 9500–9514. 10.18632/aging.103223 32427128 PMC7288956

[B115] Yuan B. Dong X. Yang F. Peng C. Wang J. Wu D. (2019a). Preparation and the antibacterial activity of AgNPs/PLGA-PEG-PLGA composite hydrogel. Chem. J. Chin. Universities-Chinese 40 (10), 2225–2232. 10.7503/cjcu20190351

[B116] Yuan H. Xu X. Feng X. Zhu E. Zhou J. Wang G. (2019b). A novel long noncoding RNA PGC1 beta-OT1 regulates adipocyte and osteoblast differentiation through antagonizing miR-148a-3p. Cell Death Differ. 26 (10), 2029–2045. 10.1038/s41418-019-0296-7 30728459 PMC6748127

[B117] Zhang G.-P. Zhang J. Zhu C.-H. Lin L. Wang J. Zhang H.-J. (2017a). MicroRNA-98 regulates osteogenic differentiation of human bone mesenchymal stromal cells by targeting BMP2. J. Cell. Mol. Med. 21 (2), 254–264. 10.1111/jcmm.12961 27860183 PMC5264139

[B118] Zhang L. Tang Y. Zhu X. Tu T. Sui L. Han Q. (2017b). Overexpression of MiR-335-5p promotes bone formation and regeneration in mice. J. Bone Min. Res. 32 (12), 2466–2475. 10.1002/jbmr.3230 PMC5732062 28846804

[B119] Zhang Y. Gao Y. Cai L. Li F. Lou Y. Xu N. (2017c). MicroRNA-221 is involved in the regulation of osteoporosis through regulates RUNX2 protein expression and osteoblast differentiation. Am. J. Transl. Res. 9 (1), 126–135. 28123639 PMC5250709

[B120] Zhang M. Liu X. Li Z. Du Y. Liu X. Lv L. (2019). Asymmetrical methyltransferase PRMT3 regulates human mesenchymal stem cell osteogenesis via miR-3648. Cell Death Dis. 10, 581. 10.1038/s41419-019-1815-7 31378783 PMC6680051

[B121] Zhang X. Wang Y. Zhao H. Han X. Zhao T. Qu P. (2020). Extracellular vesicle-encapsulated miR-22-3p from bone marrow mesenchymal stem cell promotes osteogenic differentiation via FTO inhibition. Stem Cell Res. Ther. 11 (1), 227. 10.1186/s13287-020-01707-6 32522250 PMC7285613

[B122] Zhang X. Wang W. Wang Y. Zhao H. Han X. Zhao T. (2021a). Extracellular vesicle-encapsulated miR-29b-3p released from bone marrow-derived mesenchymal stem cells underpins osteogenic differentiation. Front. Cell Dev. Biol. 8, 581545. 10.3389/fcell.2020.581545 33553139 PMC7862561

[B123] Zhang Y. Cao X. Li P. Fan Y. Zhang L. Ma X. (2021b). microRNA-935-modified bone marrow mesenchymal stem cells-derived exosomes enhance osteoblast proliferation and differentiation in osteoporotic rats. Life Sci. 272, 119204. 10.1016/j.lfs.2021.119204 33581127

[B124] Zhi F. Ding Y. Wang R. Yang Y. Luo K. Hua F. (2021). Exosomal hsa_circ_0006859 is a potential biomarker for postmenopausal osteoporosis and enhances adipogenic versus osteogenic differentiation in human bone marrow mesenchymal stem cells by sponging miR-431-5p. Stem Cell Res. Ther. 12 (1), 157. 10.1186/s13287-021-02214-y 33648601 PMC7923524

[B125] Zhong D. Xu G.-Z. Wu J.-Z. Liu H. Tang J.-Y. Wang C.-G. (2021). Circ-ITCH sponges miR-214 to promote the osteogenic differentiation in osteoporosis via upregulating YAP1. Cell Death Dis. 12 (4), 340. 10.1038/s41419-021-03586-y 33795657 PMC8016856

[B126] Zuo R. Kong L. Wang M. Wang W. Xu J. Chai Y. (2019). Exosomes derived from human CD34(+) stem cells transfected with miR-26a prevent glucocorticoid-induced osteonecrosis of the femoral head by promoting angiogenesis and osteogenesis. Stem Cell Res. Ther. 10 (1), 321. 10.1186/s13287-019-1426-3 31730486 PMC6858646

